# Selective inhibition of long isoforms of phosphodiesterase 4D mitigates liver fibrosis in mouse models

**DOI:** 10.1172/JCI182571

**Published:** 2025-11-06

**Authors:** Jeonghan Kim, Heeeun Yoon, Seoung Chan Joe, Antoine Smith, Jinsung Park, Geunhye Hong, Ji Myeong Ha, Eun Bae Kim, Ekihiro Seki, Myung K. Kim, Hae-Ock Lee, Ho-Shik Kim, Jay H. Chung

**Affiliations:** 1 Department of Biochemistry, College of Medicine and; 2 Department of Medical Sciences, Graduate School of The Catholic University of Korea, Seoul, South Korea.; 3 Laboratory of Obesity and Aging Research, Cardiovascular Branch, National Heart Lung and Blood Institute, National Institutes of Health, Bethesda, Maryland, USA.; 4 Department of Medicine, Cedars-Sinai Medical Center, Los Angeles, California, USA.; 5 Department of Microbiology, College of Medicine, The Catholic University of Korea, Seoul, South Korea.; 6 Cancer Evolution Research Center, College of Medicine, The Catholic University of Korea, Seoul, South Korea.

**Keywords:** Hepatology, Inflammation, Fibrosis, Phosphodiesterases

## Abstract

Chronic inflammation leads to tissue fibrosis, which can disrupt the function of the parenchyma of the organ and ultimately lead to organ failure. The most prevalent form of this occurs in chronic hepatitis, which leads to liver fibrosis and, ultimately, cirrhosis and hepatic failure. Although there is no specific treatment for fibrosis, the phosphodiesterase 4 (PDE4) competitive inhibitors have been shown to ameliorate fibrosis in rodent models. However, competitive inhibitors of PDE4 have shown significantly reduced effectiveness due to severe gastrointestinal side effects. The PDE4 family is composed of 4 genes (PDE4A–D), with each having up to 9 differentially spliced isoforms. Here, we report that PDE4D expression is specifically elevated during the hepatic fibrosis stage of liver disease progression. Furthermore, the expression of the long isoforms of PDE4D is selectively elevated in activated hepatic stellate cells, leading to the enhanced accumulation of extracellular matrix components. In a mouse model of liver fibrosis, genetic ablation of PDE4D or pharmacological inhibition using D159687, a selective allosteric inhibitor targeting the long isoforms of PDE4D, suppresses the expression of inflammatory and profibrogenic genes. These findings establish the long isoforms of PDE4D as key drivers of liver fibrosis and highlight their potential as therapeutic targets to ameliorate liver fibrosis.

## Abstract 2

Selective PDE4D inhibition ameliorates liver fibrosis through regulating profibrogenic and inflammatory signaling networks.

## Graphical abstract

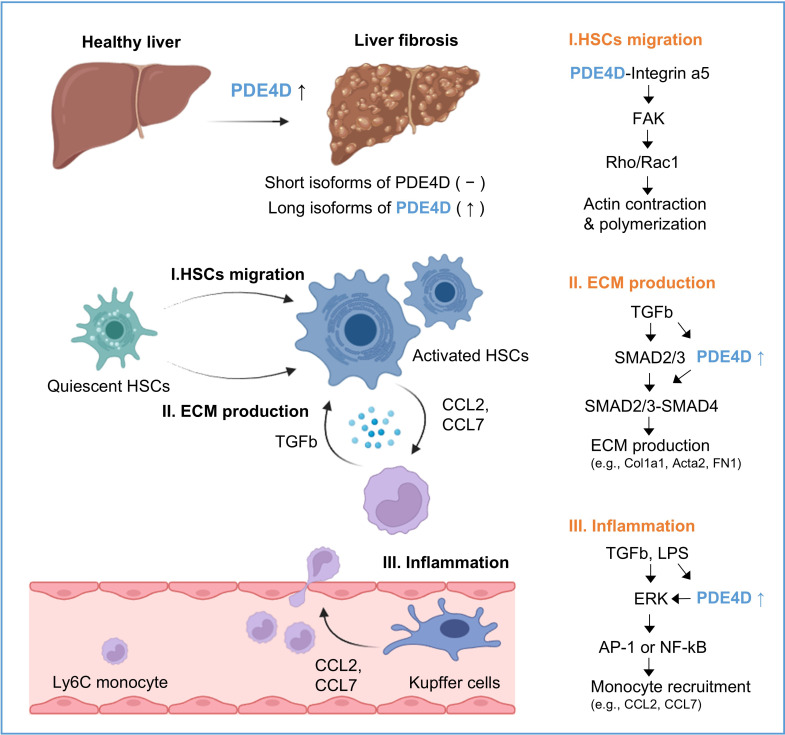

## Introduction

The prevalence of hepatitis is rising globally due to the epidemics of obesity and fatty liver disease adding to the traditional sources of liver damage, such as chronic alcoholism and viral hepatitis. Regardless of its underlying cause, liver injury in adult mammals typically triggers a wound-healing response that is invariably associated with fibrotic scarring (1, 2). This maladaptive process occurs through the replacement of the damaged parenchyma with extracellular matrix (ECM) components, such as fibrillar collagens and fibronectin (3, 4). Mild-to-moderate liver fibrosis is a reversible condition; however, excessive fibrosis can progress to an irreversible end-stage cirrhosis (3, 5, 6). Currently, liver transplantation is the only effective treatment for advanced liver fibrosis, as no approved pharmacological therapies are available (7–9).

A key driver of liver fibrogenesis is activation of hepatic stellate cells (HSCs) (10, 11), which, in a healthy liver, store vitamin A through retinyl ester-filled lipid droplets in the perisinusoidal space (10, 12). After injury, they transdifferentiate into myofibroblast-like cells, which are characterized by increased proliferation, migration, and ECM production (1, 10, 11). Another hallmark of liver fibrosis is the activation of Kupffer cells, which serve as primary sensors of liver injury (5, 12). Upon activation, these resident macrophages secrete a variety of cytokines, chemokines, and growth factors, facilitating the recruitment and activation of hepatic stellate cells, proinflammatory monocytes, and neutrophils at the injury site (13–16).

The second messenger cyclic adenosine monophosphate (cAMP) is a well-known mediator of the antifibrotic signaling and antiinflammatory response (9, 17–24). cAMP inhibits collagen synthesis by inhibiting the transforming growth factor β–induced (TGFb-induced) activation of the SMAD complex (19, 23, 25). In addition, cAMP suppresses the platelet-derived growth factor–AKT (PDGF-AKT) pathway, thereby activating forkhead box protein O1 (FOXO1), which inhibits HSCs proliferation (18, 26, 27). However, hepatic cAMP levels are decreased during liver fibrosis due to phosphodiesterases (PDEs), which hydrolyze cAMP (9, 18, 19, 23, 27). There are 11 families of PDEs (PDE1–11), with each having different physiological functions and tissue-specific expression patterns (9, 24, 28, 29). Inhibition of the PDE4 family has been shown to ameliorate metabolic and inflammatory disorders such as steatohepatitis, liver fibrosis, type 2 diabetes, and atherosclerosis in rodent models (23, 24, 30–33). Indeed, the pan-PDE4 inhibitor Roflumilast is FDA-approved for treatment of psoriasis and chronic obstructive pulmonary disease (COPD) (28). Although PDE4 inhibitors demonstrate efficacy in rodent models, their therapeutic potential in humans is substantially limited. Rodents lack an emetic reflex, whereas, in humans, optimal dosing is constrained by severe nausea and other gastrointestinal side effects at therapeutic concentrations (34–36). Therefore, selectively targeting the smallest possible subgroup of PDE4 isoforms may help minimize adverse effects while maximizing the clinical effectiveness of PDE4 inhibitors.

Here, we identified PDE4D as the key mediator among PDE4 isoforms in driving profibrogenic signaling networks that activate hepatic stellate cells (HSCs) by amplifying signaling downstream of TGFb and PDGF. Furthermore, we found that only the long isoforms of PDE4D are expressed in activated HSCs. Targeting these isoforms with D159687, a selective small-molecule inhibitor that does not trigger the emetic reflex in nonhuman primates (29, 35), effectively suppresses profibrogenic signaling and ameliorated bile duct-ligation–induced (BDL-induced) liver fibrosis. Taken together, these findings enhance our understanding of the mechanism of liver fibrosis and provide a potential therapeutic strategy against liver fibrosis.

## Results

### Specificity of PDE4D upregulation in fibrotic liver disease stages.

**Figure 1:**
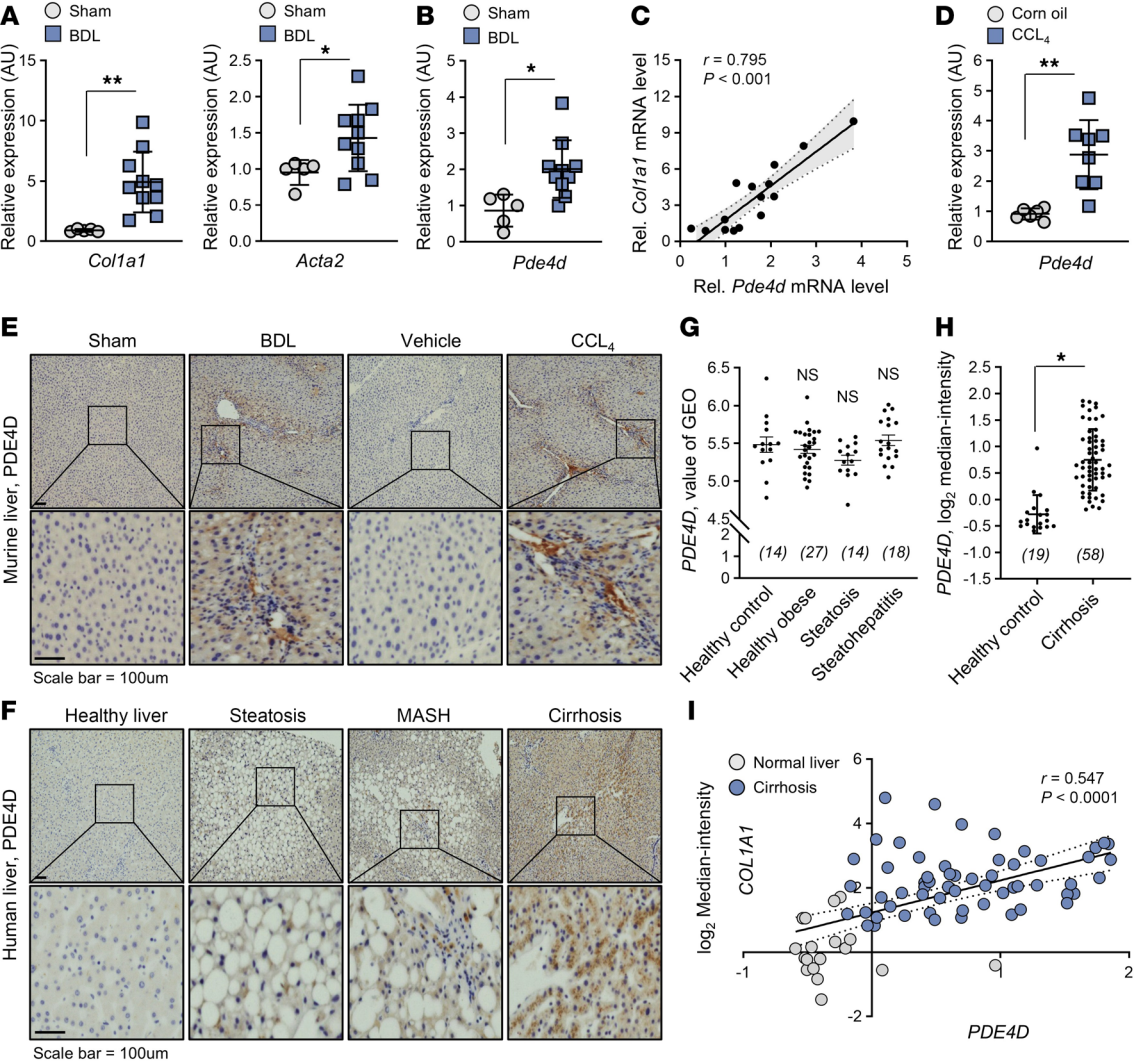
Expression levels of PDE4D in the liver disease. (**A** and **B**) The expression levels of *Col1a1*, *Acta2*, and *Pde4d* in mice (*n* = 10) with BDL-induced liver fibrosis and sham-operated controls (*n* = 5) were determined using RT-qPCR.(**C**) Correlation coefficient analysis of *Col1a1* and *Pde4d* mRNA levels. (**D**) *Pde4d* mRNA levels in mice with liver fibrosis. Mice were intraperitoneally injected with corn oil (Control) or CCl_4_ twice a week for 5 weeks. (**E**) Representative images of IHC staining for PDE4D in mouse models of liver fibrosis. A magnified image of the boxed area is shown in the bottom row. (**F**) Representative images of PDE4D-staining in liver sections from patients with various liver diseases. (**G** and **H**) Expression levels of PDE4D in various types of liver diseases. The values were derived from the GEO and Oncomine databases. (**I**) Correlation coefficient analysis of *PDE4D* and *COL1A1* mRNA levels. All values are presented as the mean ± SD. 1-way ANOVA with Tukey’s post hoc test for multiple comparisons was used for statistical analysis in **G**; A 2-tailed unpaired Student’s *t* test was used to evaluate the statistical significance in **A**, **B**, **D**, and **H**. *P < 0.05; **P < 0.01. Scale bars: 100 μl.

We induced liver fibrosis in mice by performing BDL, a commonly used model for liver fibrosis, and measured the expression levels of fibrotic markers and the four PDE4 isoforms (Supplemental Figure 1A; supplemental material available online with this article; https://doi.org/10.1172/JCI182571DS1). As expected, fibrotic markers collagen type I α 1 chain (*Col1a1*) and actin α 2, smooth muscle (*Acta2, a-Sma*) were increased in BDL mice compared with the levels in sham-operated mice (Figure 1A). The mRNA expression level of *Pde4d,* was also increased in BDL mice (Figure 1B) and was highly correlated with *Col1a1* level in the BDL mice (Figure 1C). The mRNA levels of *Pde4b* and *Pde4c* did not change, and *Pde4a* was not detectable (Supplemental Figure 1B). The *Pde4d, Col1a1,* and *Acta2* were also upregulated in carbon tetrachloride–induced (CCl_4_-induced) fibrosis (Figure 1D and Supplemental Figure 1C). Immunostaining of BDL- or CCl_4_-induced fibrotic tissue demonstrate increased PDE4D in the fibrotic lesion, which also contained activated HSCs and inflammatory cells (Figure 1E). Interestingly, PDE4D level was not changed in patients with obesity, steatosis, or steatohepatitis (Figure 1, F and G) or in obese (*ob/ob*) mice (Supplemental Figure 1, D–F) but was increased in the human cirrhotic liver (Figure 1, F and H) (37) and correlated with the levels of *COL1A1* mRNA (Figure 1I and Supplemental Figure 1G). Thus, the upregulation of PDE4D expression is specific to the fibrotic stage in the liver disease progression and may serve as a potential molecular diagnostic marker.

### Isoform-specific PDE4D expression in HSC transdifferentiation.

**Figure 2:**
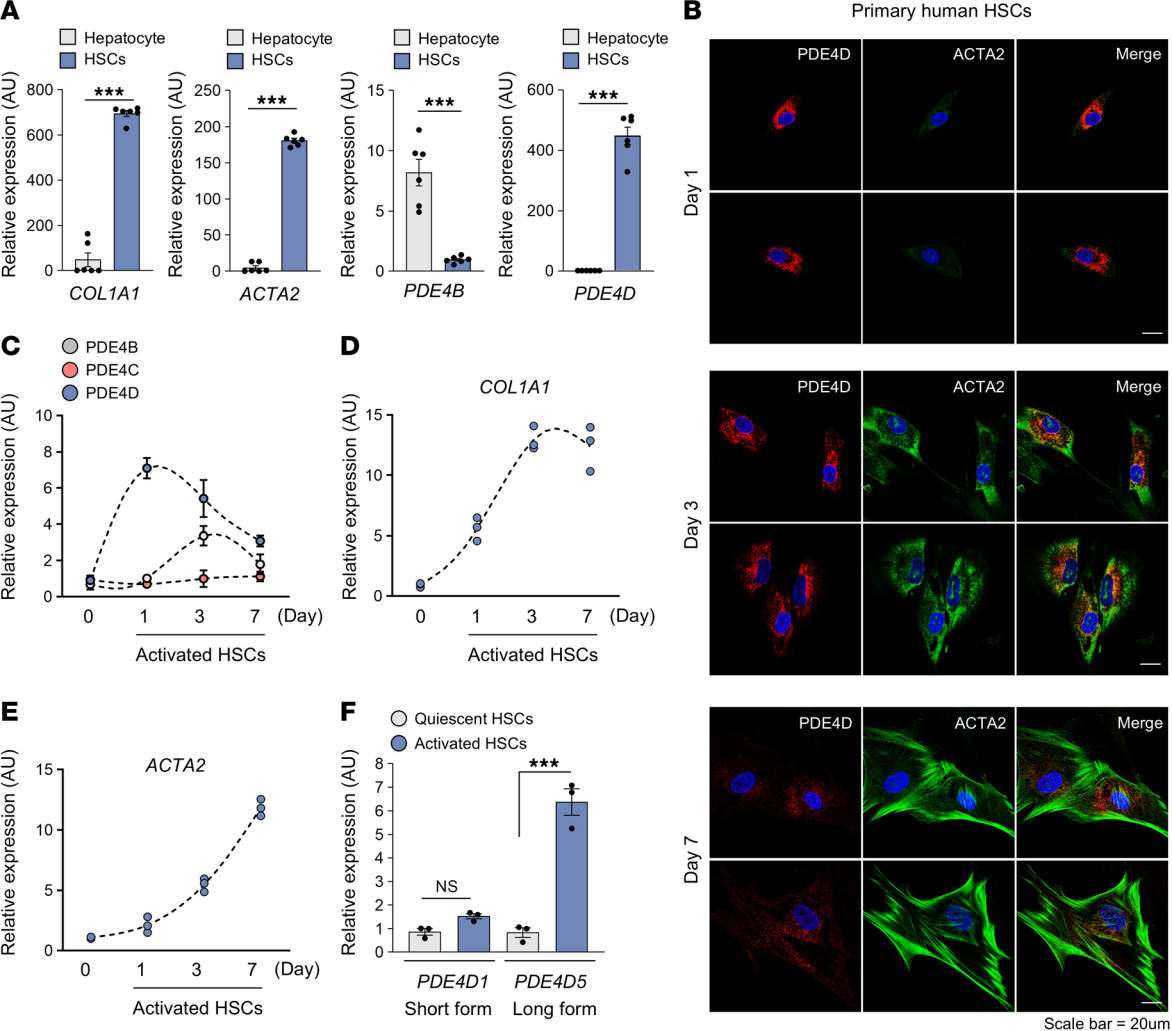
PDE4D is upregulated in culture-activated HSCs. (**A**) Comparison of *COL1A1*, *ACTA2*, *PDE4B*, and *PDE4D* mRNA levels between primary human hepatocyte and culture-activated primary human HSCs at day 1. (**B**) Culture-activated primary human HSCs were stained for PDE4D (red) and ACTA2 (green). Representative images on days 1, 3, and 7 were shown. (**C**–**E**) Comparison of *PDE4B*, *PDE4C*, *PDE4D*, *COL1A1*, and *ACTA2* mRNA levels between quiescent HSCs (day 0) and culture-activated primary human HSCs for indicated times. *PDE4A* was not detected. (**F**) The mRNA levels of the short (*PDE4D1*) and long (*PDE4D5*) isoforms were determined using RT-qPCR with specific primers at day 1. All values are presented as the mean ± SEM of at least 3 independent experiments. 1-way ANOVA with Tukey’s post hoc test for multiple comparisons was used for statistical analysis in **C**–**F**; A 2-tailed unpaired Student’s *t* test was used to evaluate the statistical significance in **A**. ***P < 0.001. Scale bars: 20 μl.

Liver damage transforms HSCs into myofibroblast-like cells with capacity for chemotaxis, contractility, and proliferation. In agreement with the histopathological pattern in the fibrotic tissues (Figure 1E), HSC markers *COL1A1* and *ACTA2* as well as *PDE4D,* but not other *PDE4* genes such as *PDE4B*, were highly expressed in primary human HSC compared with primary human hepatocytes (Figure 2A). Culturing primary HSC on a petri dish transforms them from a quiescent to an activated phenotype. The time course of the expression revealed that *PDE4D*, unlike *PDE4B* and *PDE4C*, is upregulated prior to the expression of activation markers such as COL1A1 and ACTA2 in HSCs (Figure 2, B–E). This suggests that PDE4D plays a pivotal role in the early stages of HSC transdifferentiation into myofibroblast-like cells.

PDE4D has 9 isoforms, produced by alternative mRNA splicing and/or differential promoter utilization (Supplemental Figure 1A) (38). All the isoforms have the phosphodiesterase catalytic domain in the carboxyl terminus and are classified into 3 types: long isoforms, which have the upstream conserved region (UCR)1, UCR2, and the catalytic domain; short isoforms, lacking UCR1; and (c) super-short isoforms, with no UCR1 and a truncated UCR2 domain (35, 38). Unlike many other tissues, the liver predominantly expresses the long isoforms of PDE4D (29), and the long isoform *PDE4D5* was upregulated in activated HSCs but not the short isoform *PDE4D1* (Figure 2F).

### Selective inhibition of long PDE4D isoforms prevents HSC migration.

**Figure 3:**
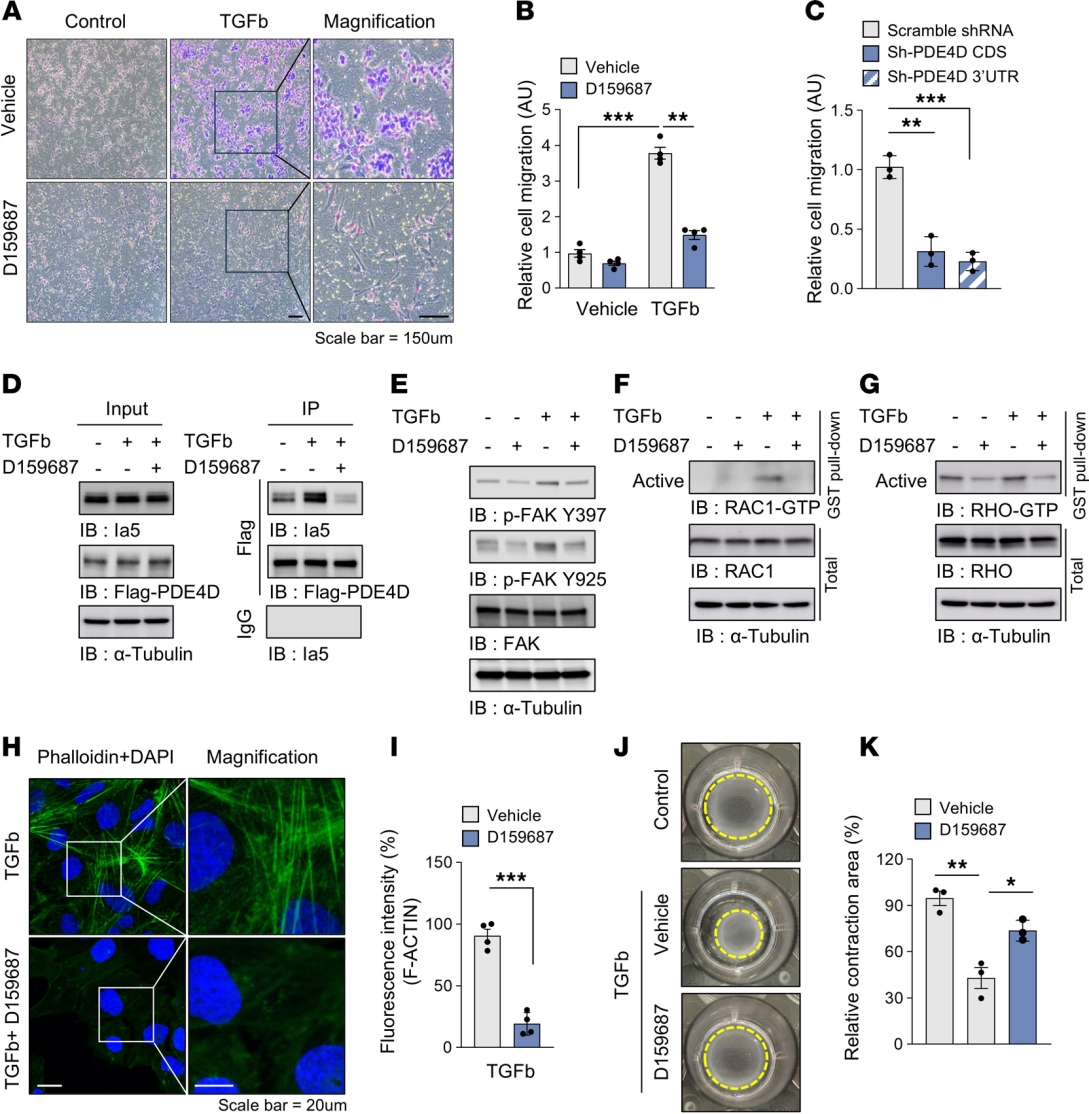
Pharmacological inhibition of PDE4D downregulates TGFb-induced HSC migration. (**A** and **B**) LX-2 transmigration assay was performed for 18 hours after treatment with 2 ng/ml TGFb with or without 10 μM D159687. (**C**) Quantification of relative cell migration in PDE4D-knockdown HSCs. (**D**) LX-2 cells stably expressing Flag-PDE4D5 (long isoform) were treated with 2 ng/ml TGFb for 24 hours in the presence of 10 μM D159687. PDE4D5 was immunoprecipitated with Flag magnetic beads and immunoblotted with anti-integrin a5. (**E**) Phosphorylated FAK was visualized using antibodies specific for Tyr397 and Tyr 925, and total FAK was used for quantitative comparison. (**F** and **G**) The levels of RAC1-GTP and RHO-GTP in LX-2 cells treated with 10 ng/ml TGFb in the presence of 10 μM D159687 for 1 hour. (**H** and **I**) Actin polymerization was visualized by staining F-actin with Alexa-Fluor-488-conjugated-phalloidin. (**J** and **K**) Representative phase contrast images of collagen-based cell contraction in LX-2 cells. All values are presented as the mean ± SEM of at least 3 independent experiments. 1-way ANOVA with Tukey’s post hoc test for multiple comparisons was used for statistical analysis in **C** and **K**; A 2-tailed unpaired Student’s *t* test was used to evaluate the statistical significance in **I**. *P < 0.05; **P < 0.01; ***P < 0.001. Scale bars: 150 μl (**A**); 20 μl (**H**).

To determine whether selective inhibition of the long isoforms of PDE4D can regulate the physiological function of HSCs, we used D159687, a specific PDE4D allosteric modulator that binds to the PDE4D UCRs and selectively inhibits the long isoforms (Supplemental Figure 1A). PDE4D expression was not changed in fatty liver (Figure 1, G and F) and D159687 did not affect lipogenesis, such as adipogenesis or palmitic acid–induced hepatic fat accumulation (Supplemental Figure 2, A–D). D159687 significantly upregulated the cAMP level in HSCs and inhibited the TGFb-induced HSC migration, a critical feature of fibrosis (Figure 3, A and B, and Supplemental Figure 3, A–C). Consistent with these findings, knocking down PDE4D expression reduced TGFb-induced migration (Figure 3C and Supplemental Figure 3D). D159687 also blocked the interaction between PDE4D and integrin a5, which promotes TGFb-mediated cell migration and transdifferentiation into myofibroblast-like cells (32, 33, 39–41) (Figure 3D). Downstream of integrin a5, phosphorylation of FAK and activation of RAC1/RHO induce cell migration through actin polymerization (40–42). These events were promoted by TGFb, but D159687 abrogated them (Figure 3, E–G). Consistent with this, D159687 inhibited the TGFb-induced actin polymerization in HSC (Figure 3, H and I) and cell contraction (Figure 3, J and K). These results suggest that the interaction of integrin a5 with the long isoforms of PDE4D may promote HSC migration through the FAK-RAC1/RHO signaling cascade (Supplemental Figure 3E).

### PDE4D enhances profibrogenic gene expression through SMAD signaling in HSCs.

**Figure 4:**
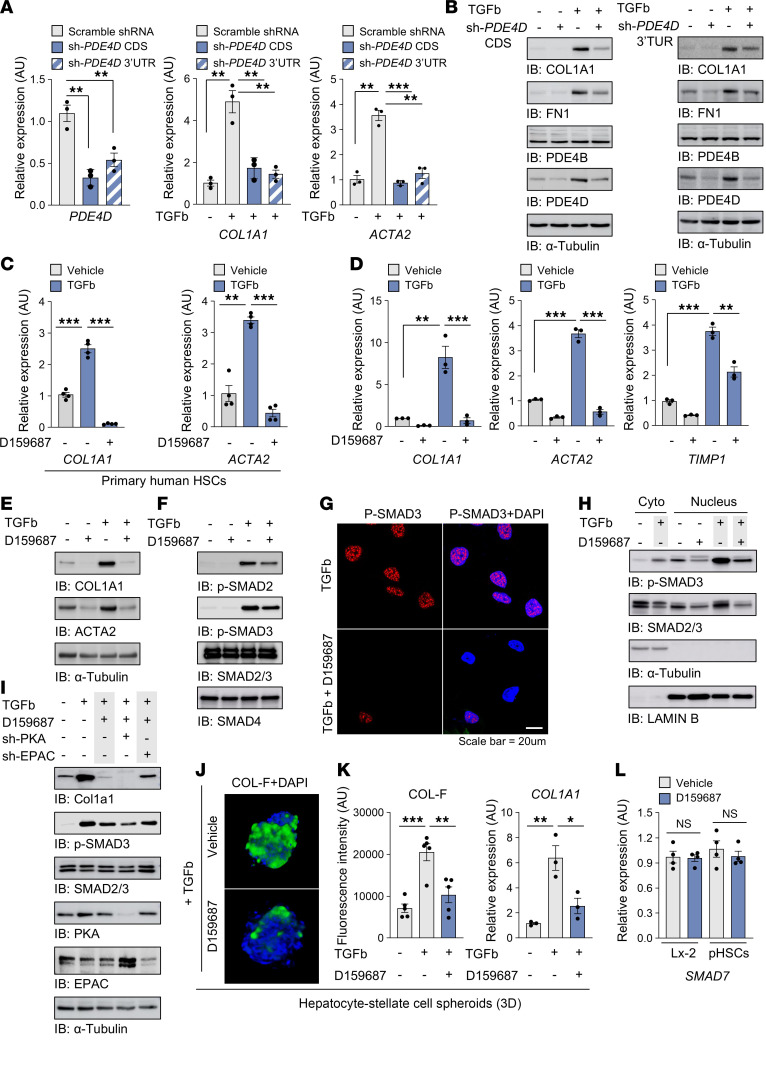
PDE4D upregulates profibrogenic genes in HSCs via regulating the SMAD signaling. (**A** and **B**) The expression levels of profibrogenic factors were determined by RT-qPCR and immunoblotting in the PDE4D knockdown cells. (**C**–**E**) Primary human HSCs and LX-2 cells were treated with 2 ng/ml TGFb for 24 hours in the presence of 10 μM D159687. The profibrogenic factors expression was determined by RT-qPCR and immunoblotting. (**F**) The phosphorylation states of SMAD2 and SMAD3 were determined using immunoblotting. (**G** and **H**) The TGFb-mediated translocation of SMAD into the nucleus was evaluated by immunostaining with anti-phospho-SMAD3 and immunoblotting after nuclear/cytoplasmic (Cyto) Fractionation. (**I**) The expression levels of COL1A1 and the phosphorylation states of SMAD3 in PKA or EPAC knockdown cells were determined by immunoblotting. (**J**) The TGFb-mediated collagen production in the primary hepatocyte-stellate cell spheroids was measured by immunostaining with collagen-binding probe, COL-F. (**K**) Collagen expression was quantified using the fluorescence intensity (left) or RT-qPCR (right). (**L**) The *SMAD7* mRNA levels in primary human HSCs and LX-2 cells treated with 10 μM D159687 for 24 hours. All values are presented as the mean ± SEM of at least 3 independent experiments. 1-way ANOVA with Tukey’s post hoc test for multiple comparisons was used for statistical analysis in **A**, **C**, **D**, **I**, and **K**; A 2-tailed unpaired Student’s *t* test was used to evaluate the statistical significance in **L**. *P < 0.05; **P < 0.01; ***P < 0.001. Scale bar: 20 μl.

TGFb is expressed in various cell types, including HSCs, during the progression of liver fibrosis and facilitates the fibrotic environment. Since PDE4D expression is upregulated during the hepatic fibrosis stage and throughout the transdifferentiation of HSCs into myofibroblast-like cells, we asked whether TGFb regulates PDE4D expression. Interestingly, the expression of Col1A1 and FN1, as well as PDE4D was upregulated by TGFb (Supplemental Figure 4A). In contrast, TGFb did not affect PDE4B expression, consistent with findings in the BDL model (Supplemental Figure 1B and Supplemental Figure 4A). To further investigate the potential role of PDE4D, we performed a proteome-wide analysis comparing WT and PDE4D knockdown cells after TGFb treatment, utilizing the Proximity Extension Assay (PEA) in combination with Next-Generation Sequencing (NGS) readout (Supplemental Figure 4, B–E). Ontology analysis and experimental validation showed that deficiency in PDE4D expression correlated with reduced extracellular structure organization, such as COL1A1 and FN1 (Figure 4, A and B, and Supplemental Figure 4, D–F). Consistent with these findings, D159687 inhibited the expression of fibrotic genes in both primary HSCs and the human hepatic stellate cell line LX-2, both before and after TGFb treatment. In contrast, A33, a PDE4B inhibitor, showed no significant effect (Figure 4, C–E, and Supplemental Figure 4, G and H). Furthermore, D159687 reduced SMAD2/3 phosphorylation and inhibited its subsequent nuclear translocation (Figure 4, F–H) and transcription of SMAD2/3-luciferase reporter gene (Supplemental Figure 4I). To elucidate the regulatory mechanism of the cAMP-SMAD pathway following PDE4D inhibition, we knocked down the effectors of cAMP signaling PKA and EPAC in LX-2 cells and analyzed fibrogenic signaling. Our findings revealed that the D159687-induced suppression of SMAD3 phosphorylation and COL1A1 expression was reversed in EPAC-knockdown LX-2 cells, indicating that D159687 inhibits fibrogenic signaling by activating EPAC rather than PKA (Figure 4I). Consistent with these findings, TGFb-induced collagen production was also inhibited in 3-dimensional hepatocyte and HSC spheroid coculture (Figure 4, J and K). Since SMAD7 modulates SMAD2/3 phosphorylation through a negative feedback loop, D159687 could reduce SMAD2/3 phosphorylation by increasing SMAD7, but D159687 did not affect SMAD7 expression in the HSCs (Figure 4L). The ECM-induced integrin–FAK signal transduction stimulates the Hippo pathway and dephosphorylation of transcription factors YAP/TAZ, leading to transcription of profibrotic genes (17, 43). Plating LX-2 cells on collagen- or fibronectin-coated plates induced phosphorylation of FAK and dephosphorylation of YAP/TAZ, but this was inhibited by D159687 (Supplemental Figure 4, J and K). Together, these findings suggest that upregulation of long isoforms of PDE4D by TGFb contributes to liver fibrosis by enhancing the expression of profibrotic genes in HSCs.

### PDE4D triggers inflammation by recruiting monocytes via CCL2 expression.

**Figure 5:**
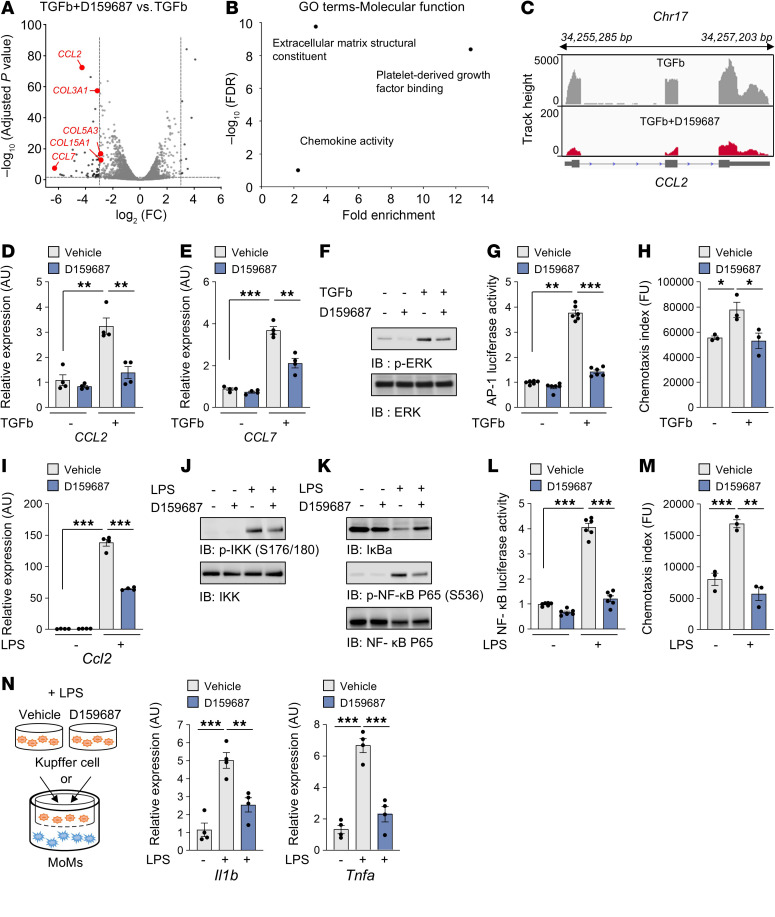
D159687 prevents monocyte recruitment by downregulating the CCL2 expression in HSCs and Kupffer cells. (**A**) A volcano plot depicting the differentially expressed genes in LX-2 cells cotreated with TGFb and D159687 versus TGFb-treated LX-2 cells. (**B**) Gene-ontology analysis of molecular functions. (**C**) Visualization of RNA-seq coverage of *CCL2*. (**D** and **E**) LX-2 cells were treated with 2 ng/ml TGFb for 24 hours in the presence of 10 μM D159687. *CCL2* and *CCL7* mRNA levels were determined via RT-qPCR. (**F**) Phosphorylated ERK was visualized by immunoblotting. (**G**) The AP-1 transcriptional activity was measured using reporter assay in AP-1-stable reporter LX-2 cells. (**H**) Effect of PDE4D on the chemotaxis of THP-1 cells in response to TGFb-mediated LX-2 CM. The migratory cells were quantified according to the fluorescence intensity. (**I**) Kupffer cells were treated with 1 μg/ml LPS in the presence of 10 μM D159687 for 24 hours. *Ccl2* mRNA levels were determined by RT-qPCR. (**J** and **K**) Phosphorylated IKK and NF-κB were visualized using antibodies specific for Phospho-IKKa/b (Ser176/180) and Phospho-NF-κB p65 (Ser536). (**L**) The NF-κB transcriptional activity in stable reporter Kupffer cells with the NF-κB response element was evaluated via a reporter assay. (**M**) Effect of PDE4D on the chemotaxis of THP-1 cells in response to LPS-mediated Kupffer cell CM. (**N**) Inflammatory cytokines, including *Il-1β* and *Tnfa*, were determined in MoMs cocultured with Kupffer cells. All values are presented as the mean ± SEM of at least 3 independent experiments. 1-way ANOVA with Tukey’s post hoc test for multiple comparisons was used for statistical analysis in **D**, **E**, **G**–**I**, and **L**–**N**. *P < 0.05; **P < 0.01; ***P < 0.001.

CCL2, also known as monocyte chemoattractant protein 1 (MCP1), plays a pivotal role in inducing local inflammatory responses during liver fibrosis by facilitating the chemotactic migration of monocytes into damaged liver tissue. Activated HSCs secrete CCL2, which not only enhances monocyte recruitment but also establishes a feed-forward loop that further stimulates HSCs activation (11, 14). Monocytes infiltrate liver tissue through CCL2-mediated signaling, where they differentiate into macrophages, particularly M2-like macrophages. These M2-like macrophages secrete elevated levels of profibrotic mediators, such as TGFb and PDGF, which further exacerbate the fibrotic process (11, 14). RNA-seq analyses indicate that D159687 treatment in LX-2 cells that were activated by TGFb modulated the mRNA expression of chemokines as well as those for ECM and PDGF signaling (Figure 5, A and B, and Supplemental Figure 5). Notably, it inhibited the expression of both *CCL2* (Figure 5, C and D) and *CCL7,* also known as monocyte chemotactic protein 3 (MCP3) (Figure 5E and Supplemental Figure 5B), which are upregulated in active HSC and cause a local inflammatory reaction during liver fibrosis (14) (Supplemental Figure 6A). The ERK and AP-1 or NF-κB pathways regulate the expression of CCL2 and CCL7 (44–46). TGFb increased ERK phosphorylation and the transcriptional activity of AP-1 in the HSC, but this was inhibited by D159687 (Figure 5, F and G); TGFb did not affect the NF-κB activity (Supplemental Figure 6, B and C). We next performed the chemotaxis assay by using the conditioned medium (CM) from the HSC culture stimulated by TGFb with or without D159687. The migration of THP-1 monocytes toward CM was significantly diminished with the CM from TGFb/D159687-treated HSC compared with that without D159687 (Figure 5H).

During liver fibrosis, the levels of endotoxins, such as lipopolysaccharide (LPS) increase in the liver, activating the Kupffer cells, the liver-resident macrophages that produce various inflammatory cytokines and chemokines (e.g., CCL2) (14, 47). D159687 downregulated LPS-induced *Ccl2* expression and activation of NF-κB in Kupffer cells (Figure 5, I–L) and suppressed monocyte migration toward the CM of LPS-treated Kupffer cells (Figure 5M). We next examined the role of PDE4D in the interaction between Kupffer cells and monocyte-derived macrophages (MoMs) by using a coculture system. Kupffer cells that were pretreated with LPS with or without D159687 were placed in the upper chambers after a change of media to remove LPS and D159687 and MoMs were placed in the bottom chamber. The inflammatory cytokines *Il1b* and *Tnfa* were upregulated in the MoMs cocultured with LPS-stimulated Kupffer cells. However, the levels of these inflammatory cytokines were reduced in MoMs cocultured with LPS-stimulated Kupffer cells that were treated with D159687 (Figure 5N). These findings suggest a mechanism whereby PDE4D increases inflammation as well as ECM production during liver fibrosis.

### PDE4D is essential for HSC proliferation and survival.

**Figure 6:**
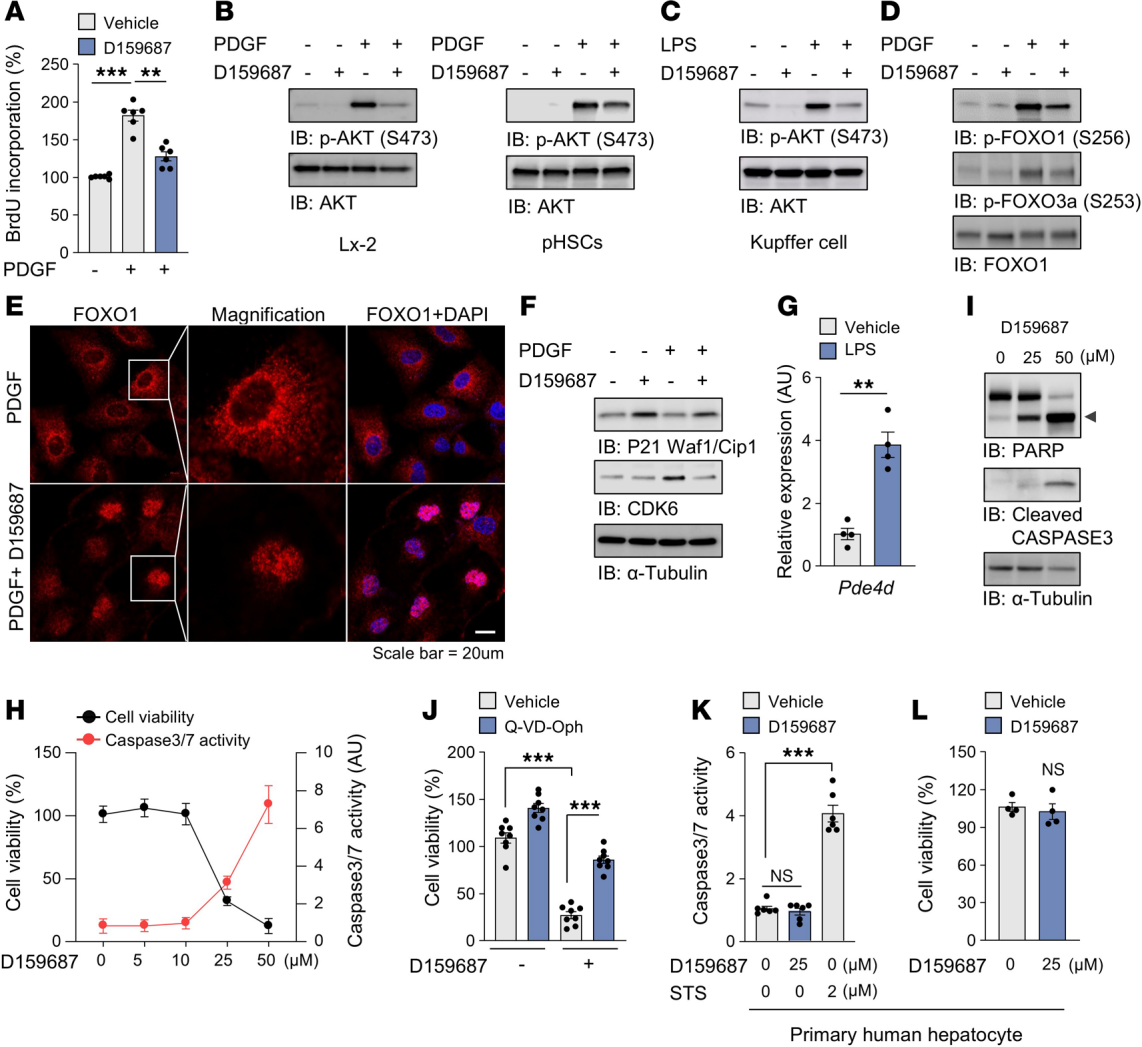
PDE4D is required for the proliferation and survival of HSCs. (A) PDGF-induced HSC proliferation (with or without D15987) was evaluated using BrdU incorporation assay. (**B**) LX-2 cells (left) and primary human HSCs (right) were treated with 10 ng/ml PDGF with or without D159687. Phosphorylated AKT was visualized using an antibody specific for ser473. (**C**) Phosphorylation of AKT in Kupffer cells. (**D**) Phosphorylation states of FOXO1 were determined by immunoblotting. (**E**) Confocal microscopy images of LX-2 cells stained with anti-FOXO1 (red) and DAPI (blue). (**F**) Expression of the cell cycle regulators. (**G**) *Pde4d* mRNA level in Kupffer cells stimulated with 1 μg LPS for 24 hours. (**H**) LX-2 Cell viability and caspase 3/7 activity under various concentrations of D159687 were evaluated. (**I**) Cleavage of PARP and caspase 3 in LX-2 cells upon D159687 treatment were evaluated by immunoblotting. (**J**) Cell viability was determined in LX-2 cells treated with D159687 in the presence of caspase inhibitor, Q-VD-Oph. (**K** and **L**) The effects of D159687 on the caspase 3/7 activity and viability of primary human hepatocytes. Staurosporine (STS, 2 μM) was used as a positive control of induction of apoptosis. All values are presented as the mean ± SEM of at least 3 independent experiments. 1-way ANOVA with Tukey’s post hoc test for multiple comparisons was used for statistical analysis in **A**, **J**, and **K**; A 2-tailed unpaired Student’s *t* test was used to evaluate the statistical significance in **G** and **L**. **P < 0.01; ***P < 0.001. Scale bar: 20 μl.

PDGF produced by resident Kupffer cells and infiltrating cells such as Ly-6C^high^ MoMs causes proliferation of HSC via paracrine mechanisms (14). D159687 suppressed the PDGF-induced BrdU-incorporation, a marker of DNA replication (Figure 6A), and the activation of the prosurvival and growth AKT signaling pathway by either PDGF (Figure 6B) or LPS (Figure 6C). Consistent with this, D159687 reduced AKT-mediated phosphorylation of Foxo1 (Figure 6D), leading to its translocation to the nucleus (Figure 6E). In the nucleus, Foxo1 likely inhibits cell growth by upregulating the expression of the cell-cycle checkpoint protein p21 and downregulating the cell-cycle regulator CDK6 (Figure 6F). Our hypothesis that PDE4D is a critical mediator of fibrosis was further supported by the observation that LPS treatment increased PDE4D expression in Kupffer cells (Figure 6G). We attempted to knockout *PDE4D* in HSCs by using CRISPR/Cas9, but most of the PDE4D knockout cells died or did not grow (data not shown), and knocking down *PDE4D* by using a short hairpin RNA also caused cell death (Supplemental Figure 7, A and B). These observations prompted us to check the effect of a higher dose of D159687 on the viability of LX-2 cells. D159687 (25 μM) decreased the survival of HSCs in a caspase 3/7-dependent manner (Figure 6, H–J) but not human primary hepatocytes (Figure 6, K and L) or Kupffer cells (Supplemental Figure 7C).

HSCs overexpress BCL2 during liver fibrosis, contributing to their resistance to apoptosis (48). To mimic this model, BCL2 was overexpressed in HSCs, which were then treated with D159687. As shown by cleaved PARP and caspase-3, apoptosis of HSCs was still induced by D159687 (25 μM), despite the overexpression of BCL2 (Supplemental Figure 7D). To explore the mechanism underlying BCL2 inhibition, the expression of BH3-only proteins involved in the regulation of apoptosis was analyzed. The results revealed that Noxa, a BCL2 inhibitor, was specifically upregulated following the treatment with D159687 (Supplemental Figure 7E). Thus, viability of HSCs is more dependent on the long isoforms of PDE4D compared with other hepatic cell types.

### Selective inhibition of long PDE4D isoforms mitigates liver fibrosis progression.

**Figure 7:**
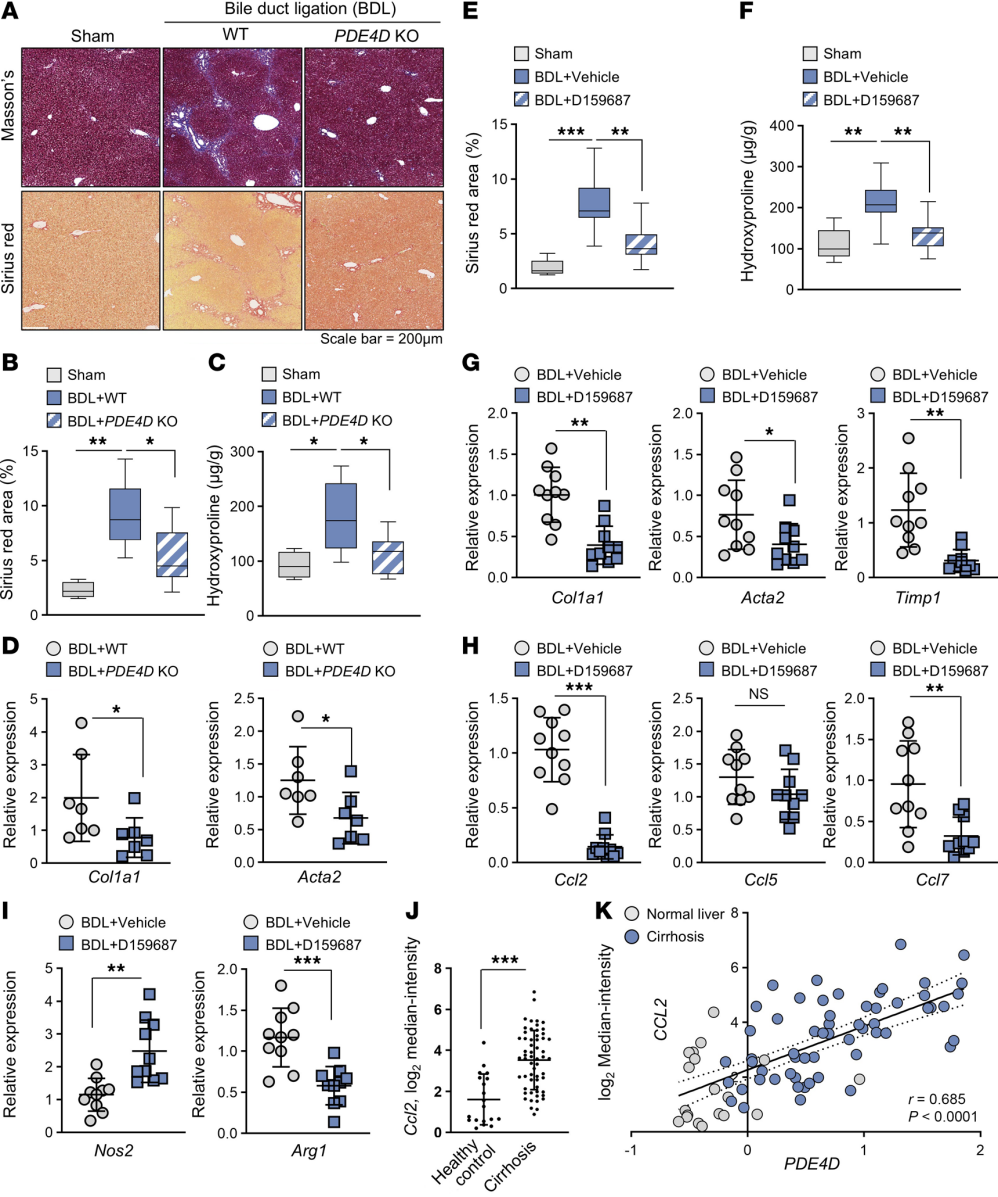
The PDE4D promotes the progression of liver fibrosis. (**A**) Representative images of mouse liver sections stained with masson’s trichrome staining or sirius red (Sham-vehicle, *n* = 4; BDL-WT, *n* = 7; BDL-PDE4D knockout, *n* = 7). (**B** and **C**) Quantification of the sirius red–positive area and the level of hydroxyproline. (**D**) The mRNA levels of the fibrotic markers *Col1a1* and *Acta2*. (**E** and **F**) BDL mice were daily treated with D159687 (3 mg/kg) or vehicle through oral gavage for 15 days after 3-day recovery from the surgery. (Sham-vehicle, *n* = 5; BDL-vehicle, *n* = 10; BDL-D159687, *n* = 10). Quantification of the Sirius red–positive area and the level of hydroxyproline. (**G**–**I**) The mRNA levels of the fibrotic markers *Col1a1*, *Acta2*, and *Timp1*; chemokines *Ccl2*, *Ccl5*, and *Ccl7*; and the macrophage polarization markers *Nos2* and *Arg1*; in the livers of the mice were determined using RT-qPCR. (BDL-vehicle, *n* = 10; BDL-D159687, *n* = 10). (**J**) Hepatic *CCL2* mRNA levels in healthy individuals and patients with cirrhosis. The values were derived from the Oncomine databases. (**K**) Correlation coefficient analysis of *CCL2* and *PDE4D* mRNA levels in cirrhosis patients. All the values are presented as the mean ± SD. 1-way ANOVA with Tukey’s post hoc test for multiple comparisons was used for statistical analysis in **B**, **C**, **E**, and **F**; A 2-tailed unpaired Student’s *t* test was used to evaluate the statistical significance in **D** and **G**–**J**. *P < 0.05; **P < 0.01; ***P < 0.001. Scale bar: 200 μl.

We performed BDL in both WT and PDE4D-knockout mice to determine the role of PDE4D in liver fibrosis. In liver tissues of WT mice, liver fibrosis had developed in BDL-operated mice compared with sham surgical control. However, in the PDE4D-knockout mice, there was a significant decrease in collagen fiber accumulation — as demonstrated by reduced Sirius red or Masson’s trichrome staining (Figure 7, A and B) — lower hydroxyproline content (Figure 7C), and diminished expression of fibrogenic genes (Figure 7D). We next evaluated the antifibrotic efficacy of D159687 in BDL. Consistent with this, daily oral administration of D159687 reduced collagen accumulation and the expression of fibrogenic genes (Figure 7, E–G, and Supplemental Figure 8A) as well as chemokines Ccl2 and Ccl7, but not Ccl5 (Figure 7H).

To investigate whether D159687 mitigates hepatic fibrosis in another model as well as to demonstrate its efficacy in ameliorating fibrosis after it has started, we employed the choline-deficient, L-amino acid–defined high-fat diet (CDAHFD) model. Administration of D159687 after 10 weeks on CDAHFD, after fibrosis has already been established, reduced collagen accumulation and the expression of fibrogenic genes (Supplemental Figure 8, B–E) as well as chemokines Ccl2 and Ccl7, but not Ccl5 (Supplemental Figure 8F). More importantly, it suppressed fibrosis progression, demonstrating its therapeutic as well as preventive potential. Interestingly, D159687 did not affect CDAHFD-induced steatosis, but this is consistent with the observation that PDE4D expression is not increased in steatosis (Figure 1, F and G, and Supplemental Figure 8G).

The mechanism by which macrophage polarization affects liver fibrosis is complex and could differ depending on the context. M1-like macrophages have been reported to ameliorate liver fibrosis by recruiting the Ly-6C^low^ restorative macrophages, which secrete matrix metalloproteinases into the fibrotic liver tissue (49). Interestingly, the M1-like macrophage marker nitric oxide synthase 2 (Nos2) was upregulated in the D159687-treated liver, whereas the M2-like macrophage marker Arginase 1 (Arg1) was downregulated (Figure 7I). Supporting our hypothesis that PDE4D promotes inflammatory cell infiltration, *CCL2* levels in the livers of patients with cirrhosis were significantly increased compared with healthy individuals and correlated with *PDE4D* levels (Figure 7, J and K). These results suggest that inhibition of the long isoforms of PDE4D arrests the progression of liver fibrosis by suppressing profibrogenic and inflammatory signaling networks in both HSC and Kupffer cells (Supplemental Figure 9).

## Discussion

Fibrosis and the accompanying deposition of collagen are central components of wound healing (1, 10). In organs such as skin, fibrosis restores the structural integrity and the barrier function that was compromised by injury (1). However, in vital organs such as the liver, kidney, heart, and lungs, chronic inflammation and the resulting fibrosis can lead to irreversible replacement of parenchymal cells with collagen, leading to diseases such as cirrhosis in the case of the liver and COPD in the case of the lungs (6, 10). PDE4 inhibitors have been approved by the FDA for indications such as COPD and psoriasis (31, 36). They are competitive inhibitors of the catalytic domain, which is highly conserved in all four PDE4s; as a result, they are pan-PDE4 inhibitors (9, 28). The UCRs are less conserved between the 4 PDE4s and therefore pose an ideal targeting site for development of an allosteric inhibitor with a greater selectivity and, therefore, safety at therapeutic doses (28, 31, 35). PDE4D variants are composed of variable N-terminal sequences, UCR, and a catalytic domain. Long isoforms of PDE4D contain both UCR1 and UCR2 regulatory domains. UCR1 interacts with UCR2 and the catalytic domain, helping to stabilize the structural framework of long isoforms (35). This interaction ensures that UCR2 can adopt the closed conformation needed for effective inhibition by D159687 (35). The UCR2 domain interacts with specific inhibitors, D159687, making it essential for their activity. Short and super-short isoforms lack UCR1 or have truncated UCR2 domains, which drastically diminished their interaction with D159687 (35). Thus, D159687 specifically inhibits the long isoforms of PDE4D, improving cognition without inducing the emetic reflex in nonhuman primates. (28, 35). Fortuitously, the long isoforms of PDE4D are also the predominant isoforms of PDE4D in the liver and are specifically induced during the activation of HSCs. Taking advantage of these converging properties, we used D159687 to investigate the role of the long isoforms of PDE4D in liver fibrosis.

HSCs, which make up approximately 10% of all resident liver cells, are the major fibrogenic cell and play critical roles in both the initiation and perpetuation of fibrosis (11). As such, they engage in an extremely complex interplay with the damaged hepatocytes, immune cells, and microenvironment that ultimately results in ECM accumulation (1, 10, 25). We found that D159687 blunted the HSC response to the migratory, proliferative, fibrogenic, and inflammatory pathways activated by extracellular signaling molecules such as TGFb and PDGF. This was accomplished, in part, by suppressing the key downstream transcription factor SMAD, which regulates the expression of collagen and actin, and of AP-1, which is activated by ERK and regulates the expression of *CCL2* and *CCL7*. D159687 may also affect the HSC function by other indirect pathways such as activation of AMP-activated kinase (50), which promotes autophagy and oxygen radical scavenging (5, 51). Pathogen-associated molecular patterns (PAMPs), such as LPS and bacterial DNA, stimulate the progression of liver fibrosis (14, 21, 52). Following liver injury, lipopolysaccharide (LPS) derived from intestinal microbiota can enter the liver via the portal vein, where it activates Kupffer cells. These activated Kupffer cells then secrete chemokines, such as CCL2, which recruit monocytes to the site of injury (14, 21, 47). D159687 blocked the LPS-induced increase in NF-κB transcriptional activity, thereby preventing *Ccl2* expression in Kupffer cells. The recruited circulating monocytes infiltrate into the injured site, where CCL2 is produced. MoMs accelerate the inflammatory response by interacting with Kupffer cells. In our coculture model of Kupffer cells and MoMs, D159687 prevented the induction of expression of MoM inflammatory cytokines, such as *Il1b* and *Tnfa*, by LPS-stimulated Kupffer cells. It is surprising that just one subclass of PDE4D (i.e., long isoforms) regulates so many aspects of HSC and Kupffer cell function.

PDE4 isoforms have widespread distributions across various tissues and overlapping tissue expressions, but they serve distinct physiological roles in different cellular compartments (9, 53, 54). At the cellular level, the absence of PDE4B leads to increased levels of basal and hormone-dependent cAMP in a pool near the plasma membrane, while depletion of PDE4D results in global cAMP accumulation, including at the plasma membrane (54). In addition, PDE4B shows higher expression levels in the lung compared with PDE4D and has been implicated in the development of pulmonary fibrosis, whereas PDE4D expression is elevated in the liver under pathological conditions, as shown in this study, thereby promoting liver fibrosis (53). Thus, understanding the specific tissue expression and functional roles of these phosphodiesterases is crucial for developing targeted therapies without undesired systemic effects.

There has been considerable effort to develop safe and effective treatments that directly target the key effectors of HSC function in liver fibrosis, including agonists of peroxisome proliferator–activated receptors (11, 25). However, their benefit-to-risk ratio remains to be determined in a large clinical trial. Our study advances the search for liver fibrosis treatment by identifying a target as the long isoforms of PDE4D. Although it was not the focus of this study, the molecular and cellular mechanisms that drive fibrogenesis in other tissues (e.g., pulmonary fibrosis) overlap with those in the liver (10, 24, 25). Therefore, targeting the long isoforms of PDE4D may be effective for treating other fibrotic diseases as well as liver fibrosis (55).

## Methods

### Sex as a biological variable.

Our study examined male and female mice, and findings were similar for mice of both sexes.

### Animal models of liver fibrosis.

Bile duct ligation (BDL) was performed in 12-week-old C57BL/6 mice (*n* = 20) at the Animal Surgery and Resources Core Facility in NHLBI to establish an animal model of liver fibrosis. The mice were anesthetized with isoflurane (3%) delivered through a gas anesthesia machine vaporizer. A ventral midline abdominal incision was then made, and the skin was dissected free from the underlying muscle fascia and reflected laterally. The incision was then extended through the abdominal wall. A ligature was placed around the common bile duct (cranial to the pancreatic duct junction and caudal to the bile ducts from each liver lobe) and then secured to occlude the duct. The abdominal wall was closed with an absorbable suture. Bupivacaine (up to 2 mg/kg) was administered along the incision for pain relief. The skin was closed with surgical staples. A sham operation was performed as above, omitting the BDL in control mice (*n* = 5). BDL mice were treated after 3 days of recovery from surgery with freshly diluted D159687 (3 mg/kg) or vehicle by oral gavage daily for 15 days. For the diet-induced MASH and liver fibrosis model, mice were maintained on a CDAHFD for a total of 12 weeks. From week 10 to week 12, freshly diluted D159687 (3 mg/kg) or vehicle was administered daily by oral gavage. PDE4D knockout mice were obtained from Marco Conti (University of California San Francisco, San Francisco, California, USA). Liver tissue from carbon tetrachloride (CCl4)-injected mice was obtained from Ekihiro Seki (Cedars-Sinai Medical Center). Mice were injected intraperitoneally with CCl4 (0.5 ml/kg; 1:4 dilution in corn oil) (Sigma-Aldrich) every 3 days for a total of 10 injections to establish the CCl4 model. Control mice received corn oil. Histology procedures were performed at the NIH/NHLBI Pathology Core Facility and Histoserve.

### Cell culture and stable cell line generation.

Human hepatic stellate cells (LX-2 cell line; Millipore Sigma) were cultured in complete Dulbecco’s modified Eagle’s medium (DMEM; Corning) supplemented with 2% fetal bovine serum (FBS; Sigma-Aldrich) and 1% penicillin and streptomycin antibiotics (Gibco) at 37°C with 5% CO_2_. Primary human hepatic stellate cells (Lonza or Zenbio) were grown in collagen-coated cell culture plates in a stellate cell growth medium (Lonza or Zenbio). Primary human hepatocytes (Zenbio) were cultured in hepatocyte maintenance medium (Zenbio) following the manufacturer’s instructions. A murine Kupffer cell line was grown in collagen-coated cell culture plates with RPMI-1640 medium supplemented with 10% fetal bovine serum (FBS; Sigma-Aldrich) and 1% penicillin and streptomycin antibiotics (Gibco).

LX-2 cells were transduced by Mission shRNA lentiviral transduction particles to knock down the expression of the phosphodiesterase 4D (PDE4D) gene (Supplemental Table 1). Control LX-2 cells were transduced with Mission shRNA control transduction particles in the presence of polybrene. Transduced cells were selected with 3 μg/ml puromycin. LX-2 cells and Kupffer cells expressing a luciferase reporter gene were established using lentiviral transduction particles encoding NF-κB response elements (Kerafast) and AP-1 response elements (Gentarget) and selected with 5 μg/ml puromycin. Luciferase activity was expressed relative to the total protein concentration of cell lysates. Most cells in experiments evaluating pharmacological PDE4D inhibition were pretreated with D159687 in DMEM with 0.2% FBS for 24 hours. Cells were tested for mycoplasma infection before each experiment.

### RNA-seq and bioinformatic analyses.

RNA-seq was carried out as previously described, with minor modifications (48). LX-2 cells were pretreated with 10 μM D159687 in DMEM with 0.2% FBS for 24 hours and then stimulated with 2 ng/ml TGFb for 24 hours. Total cellular RNA was isolated using the RNeasy RNA extraction kit (QIAGEN). RNA integrity was verified by an Agilent Bioanalyzer. RNA-seq was performed in the NIH DNA Sequencing and Genomics Core Facility. A TruSeq stranded total RNA library preparation kit (Illumina) was used to construct RNAseq libraries, according to the manufacturer’s instructions. The resulting libraries were quantified by a QuBit fluorometer (Thermo Fisher) and sequenced on a Hiseq-3000 sequencer with a 2×50 bp modality.

RNA-seq analysis was performed in the NIH-Bioinformatics and Computational Biology Core Facility. The quality of the sequencing reads was assessed with FastQC (v0.11.9). Reads with adapter contamination were trimmed using Trimmomatic (v0.39), and the remaining reads were aligned to a human genome reference sequence (GRCh38) with STAR (v2.7.3a) using the default settings. Gene counts were generated using featureCounts from Subread (v2.0.0). Differentially expressed genes were identified with DESeq2 (v1.30) R package and GO enrichment analysis was performed with the clusterProfiler R package (v3.18). The false discovery rate (FDR) was calculated using the Benjamini–Hochberg algorithm.

### Extraction of total RNA and RT-qPCR.

Total RNA was extracted using the RNeasy Mini Kit (Qiagen), according to the manufacturer’s instructions. The first-strand cDNA was synthesized from 1 μg purified mRNA using the AccuPower CycleScript RT PreMix (BioNeer). Samples were processed with 12 cycles of 30 seconds at 20°C, 4 minutes at 48°C, 30 seconds at 55°C, and then heat inactivated by 94°C for 5 minutes. RT-qPCR was carried out to analyze the mRNA level using the LightCycler 96 system (Roche Life Science) with SYBR Green master mix (Roche) and related primers (Supplemental Table 2). To design primers that specifically amplify the short form (PDE4D1) and the long form (PDE4D5) of PDE4D without cross reactivity, we targeted unique, nonoverlapping regions of each isoform. The ratio of the target gene expression to GAPDH expression (internal mRNA standard) was calculated using LightCycler 96 software (Roche). The quality of the RT-qPCR results was evaluated by melting curve analysis.

### Preparation of cell lysates and immunoblot analyses.

Cells were washed twice with ice-cold PBS and harvested in RIPA buffer (Millipore Sigma) with PhosSTOP phosphatase inhibitors (Roche) and cOmplete protease inhibitors (Roche). Nuclear and cytoplasmic extracts were obtained using NE-PER Nuclear and Cytoplasmic kits (Pierce), according to the manufacturer’s instructions. The total protein concentration was measured by a Coomassie Plus protein assay (Thermo Fisher) and subjected to immunoblotting. Primary antibodies were used at 1:1,000 dilution, including COL1A1 (#84336, Cell signaling); ACTA2 (#A2547, Sigma-Aldrich); COL1A1 (#84336, Cell signaling); p-SMAD2 (#3108, Cell signaling); p-SMAD3 (#ab52903, abcam); SMAD2/3 (#8685, Cell signaling); SMAD4 (#46535, Cell signaling); LAMIN (#13435, Cell signaling); p-ERK (#4370, Cell signaling); ERK (#4696, Cell signaling); p-IKK (#2697, Cell signaling); IKK (#11930, Cell signaling); p-NF-κB p65 S536 (#3033, Cell signaling); NF-κB P65 (#8242, Cell signaling); p-AKT (#4060, Cell signaling); AKT (#4691, Cell signaling); p-FOXO1 S256 (#9461, Cell signaling); p-FOXO3a S253 (#13129, Cell signaling); FOXO1 (#2880, Cell signaling); p18 INK4C (#2896, Cell signaling); p21 Waf1/Cip1 (#2947, Cell signaling); CDK4 (#12790, Cell signaling); CDK6 (#3136, Cell signaling); PARP (#9542, Cell signaling); CASPASE3 (#14220, Cell signaling); Integrin a5 (#ab150361, abcam); p-FAK Y397 (#8556, Cell signaling); p-FAK Y925 (#3284, Cell signaling); FAK (#13009, Cell signaling); FLAG (#F1804, Sigma-Aldrich); a-tubulin (sc-8035, Santa Cruz).

### Collagen-based cell contraction assays.

LX-2 cells were pretreated with or without 10 μM D159687 in DMEM with 0.2% FBS for 24 hours. Cells were harvested and resuspended in 500 μl of the collagen solution provided with the cell contraction assay kit (Cell Biolabs Inc). Samples were plated into 24-well plates and incubated at 37 °C with 5% CO_2_ for 1 hour to allow the collagen to polymerize. Culture media containing 0.5 ng/ml TGFb and either vehicle or 10 μM D159687 was added to each well, and the cells were incubated for 2 days. The culture medium was changed daily, and free-floating gels were imaged by GEL imager (GE AI600).

### Cell migration assays.

Chemotaxis assays were performed using the CytoSelect Cell Migration Kit (5 μm, fluorometric format; Cell Biolabs). The lower wells in the feeder tray were filled with 150 μl LX-2 conditioned medium, and the upper wells in the membrane chamber (5 μm) were filled with THP-1 cells (2 × 10^5^ cells) in serum-free medium (100 μl) and incubated for 2 hours at 37°C with 5% CO_2_. The medium containing THP-1 cells from the top side of the membrane chamber was then removed and placed into a cell harvesting tray containing 150 μl of cell detachment solution. Lysis buffer/CyQuant GR dye solution was added to the cell detachment solution and incubated for 20 minutes at room temperature. The migratory cells were quantified by fluorescence intensity at 480 nm/520 nm.

Scratch-wound migration assays were performed on confluent LX-2 cells using a 10-μl pipette tip to introduce a wound. The plates were then incubated with 5 μM D159687 in the presence of TGFb for 36 hours. Migration was measured by quantifying the cell-free area of the wound.

### Hepatocyte-stellate cell spheroids assays.

Spheroid-formation assays were performed to mimic the cellular complexity of the liver. Hepatocyte-stellate cell-spheroid cultures were produced using primary human hepatocytes, primary hepatic stellate cells, and a 3D Hepatocyte-Stellate Cell Spheroids kit (Sciencell) and resuspended 3D culture medium (Sciencell). Spheroid suspensions were incubated at 37 °C with 5% CO2 for 16 hours. Spheroids were pretreated with or without 10 μM D159687 in DMEM with 0.2% FBS for 24 hours and then stimulated with 5 ng/ml TGFb for 2 days. Spheroids were stained with 2 μM COL-F for 30 minutes to determine collagen levels. After washing with a live cell imaging solution (Invitrogen), spheroids were observed using an LSM880 confocal microscope (Zeiss).

### Cell viability and caspase 3/7 activity.

Cell viability was measured by a colorimetric viability assay kit for cholecystokinin octapeptide (CCK-8; Dojindo). Caspase activity was determined using a Caspase-Glo 3/7 luminescent assay kit (Promega) or by immunoblotting using CASPASE3 antibodies. Absorbance and luminescence were measured using a microplate reader (Cytation 5, BioTek). For live/dead cell staining, cells were stained with the acetoxymethyl ester of calcein (calcein-AM) for live cell staining and ethidium homodimer-1 (EthD-1) for dead-cell staining. The stained LX-2 cells were visualized using an LSM880 confocal microscope equipped with a 63X/1.4 Plan-Apochromat lens (Zeiss).

### Statistics.

Statistical comparisons between groups were performed by 2-tailed unpaired Student’s *t* test and ANOVA with Tukey’s post hoc test for multiple comparisons (GraphPad Prism version 9 software). All values are presented as the mean ± standard error of the mean (SEM) of at least three independent experiments, and differences were considered statistically significant at *P* < 0.05.

### Study approval.

All mouse experiments were approved by the Institutional Animal Care and Use Committee of the NIH/NHLBI.

### Data and materials availability.

All data associated with this study are present within the main text in the paper or in the Supporting Data Values file. The database of human cirrhosis patient is derived from the Oncomine (PDE4D; Mas Liver). Gene Expression Omnibus (GEO) database of human obesity patients is supported by National Center for Biotechnology Information (GEO accession number: GSE48452).

## Author contributions

JK supervised and performed most of the experiments, analyzed the data, interpreted results, and wrote the manuscript. HY, SCJ, JP, AS, GH, JMH, EBK, MKK, HOL, and HSK helped with experiments. ES provided the liver tissue samples of CCl_4_-injected mice. JHC supervised the study, analyzed the data, and revised the manuscript.

## Funding support

This work is the result of NIH funding, in part, and is subject to the NIH Public Access Policy. Through acceptance of this federal funding, the NIH has been given a right to make the work publicly available in PubMed Central.

- The Intramural Research Program of the National Heart Lung and Blood Institute.
- The National Research Foundation of Korea (NRF) grant funded by the Korea government (MSIT) (No. RS-2023-00210934, No. RS-2023-00265380, No. RS-2023-00220840, RS-2025-16069430, and NRF-2019R1A2C1008251).
- The NIH/NIDDK (R01DK085252 to ES).

## Supplementary Material

Supplemental data

Unedited blot and gel images

Supporting data values

## Version 1 11/06/2025

In-Press Preview

## Version 2 01/02/2026

Electronic publication
