# Supplementary material for: Selective inhibition of long isoforms of phosphodiesterase 4D mitigates liver fibrosis in mouse models
Source: J Clin Invest. 2025 Nov 6;136(1):e182571. doi: 10.1172/JCI182571 (PMC12721886; doi:10.1172/JCI182571)
Supplement: Supplemental data [file jci-136-182571-s213.pdf]

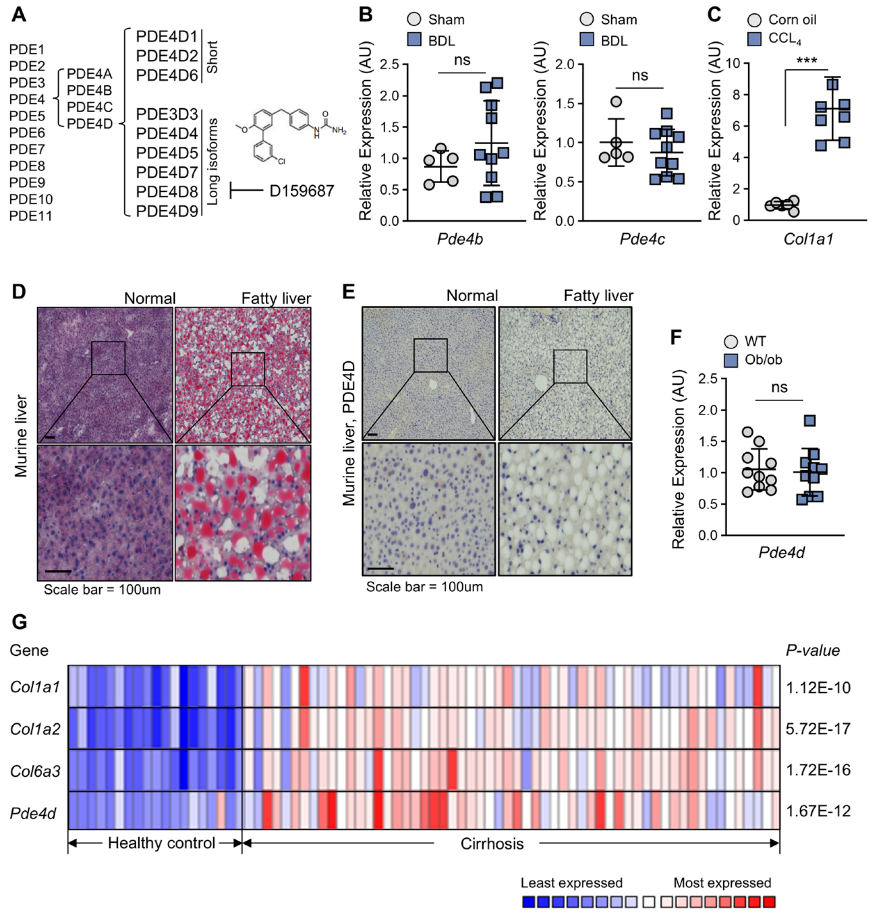

Supplemental Figure 1. Expression level of PDE4D in the fatty or fibrotic liver. (A) PDE isoforms (left) and Chemical structure of D159687 (right), an allosteric inhibitor of PDE4D long isoforms. **(B, C)** *Pde4b* and *Pde4c* mRNA levels in BDL-treated (n = 10) and sham-operated (n = 5) mice. The *Col1a1* and *Acta2* mRNA levels in CCL4-treated (n = 8) and corn oil-treated (n = 6) mice were determined using RT-qPCR. **(D, E)** Representative images of liver tissues stained with Oil RED-O (ORO) and anti-PDE4D. **(F)** The hepatic *Pde4d* mRNA levels in mice with fatty livers [C57BL/6J-WT, n = 10; B6.Cg-Lep^{ob}/J (ob/ob), n = 10]. **(G)** Expression patterns of collagen isoforms and PDE4D in healthy individuals and patients with cirrhosis. The values were derived from the Oncomine database. All values are presented as the mean ± SD. A two-tailed unpaired

Student's t-test was used to evaluate the statistical significance in A, B and E. $^{**}p < 0.01$; $^{***}p < 0.001$; ns, not significant.

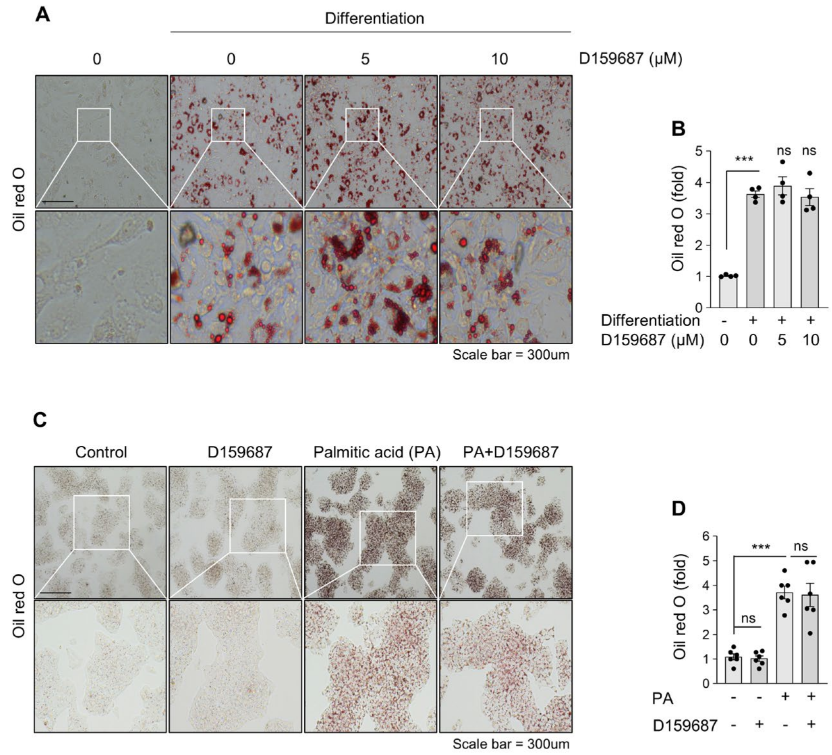

Supplemental Figure 2. Effects of D159687 on lipid accumulation. (A) Fully confluent 3T3-L1, a preadipocyte cells were pretreated with the indicated dose of D159687 for 24 hours. The cells were incubated with the differentiation-medium for 3 days and then treated with the maintenance medium containing D159687 for 2 days. The adipocyte differentiation level was assessed via ORO staining. **(B)** Quantification of the ORO-stained lipid droplets. **(C)** HepG2 cells were pretreated with 10 μM D159687 for 24 hours and incubated with BSA-conjugated palmitic acid (400 μM) for 24 hours. The lipid accumulation was visualized by ORO staining. **(D)** Quantification of the ORO-stained lipid droplets. All values are presented as the mean ± SEM of at least three independent experiments. One-way ANOVA with Tukey's post-hoc test for multiple comparisons was used for statistical analysis in B and D; ***p < 0.001; ns, not significant.

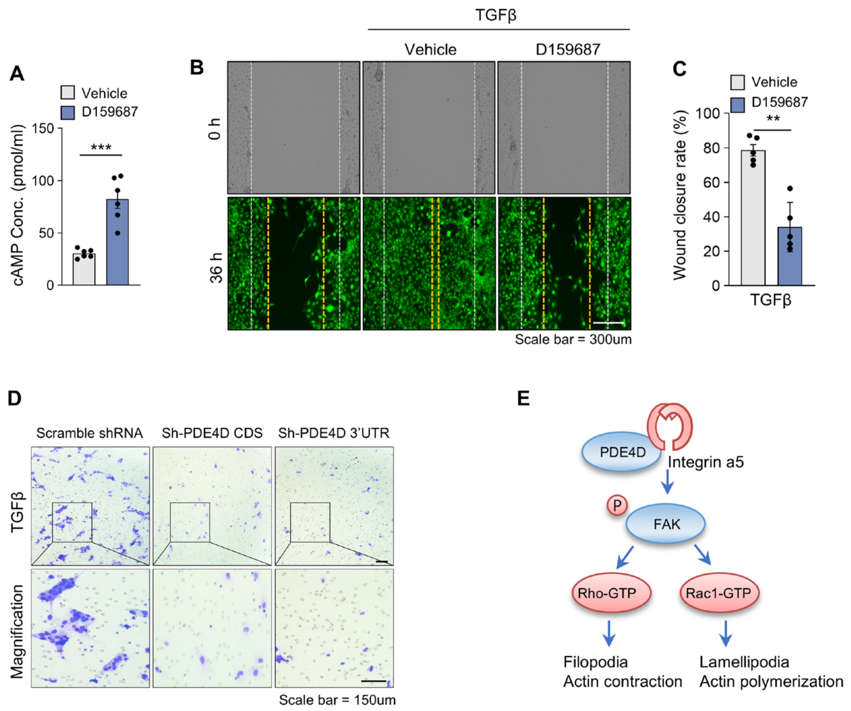

Supplemental Figure 3. PDE4D mediated HSC migration. (A) Intracellular cAMP levels in D159687-treated LX-2 cells. Cells were treated with 10 µM D159687, and intracellular cAMP was measured. **(B)** The scratch-wound migration assay was performed on LX-2 cells treated with D159687 and TGFβ for 36 hours. **(C)** Quantification of the wound closure rate. **(D)** LX-2 transmigration assay was performed for 18 hours after treatment with of 2 ng/ml TGFβ in the PDE4D knockdown HSCs. **(E)** A schematic diagram showing the signaling pathway that modulates the TGFβ-induced migration of LX-2 cells through PDE4D. All values are presented as the mean ± SEM of at least three independent experiments. A two-tailed unpaired Student's t-test was used to evaluate the statistical significance in A, C, and E. **p < 0.01; ***p < 0.001.

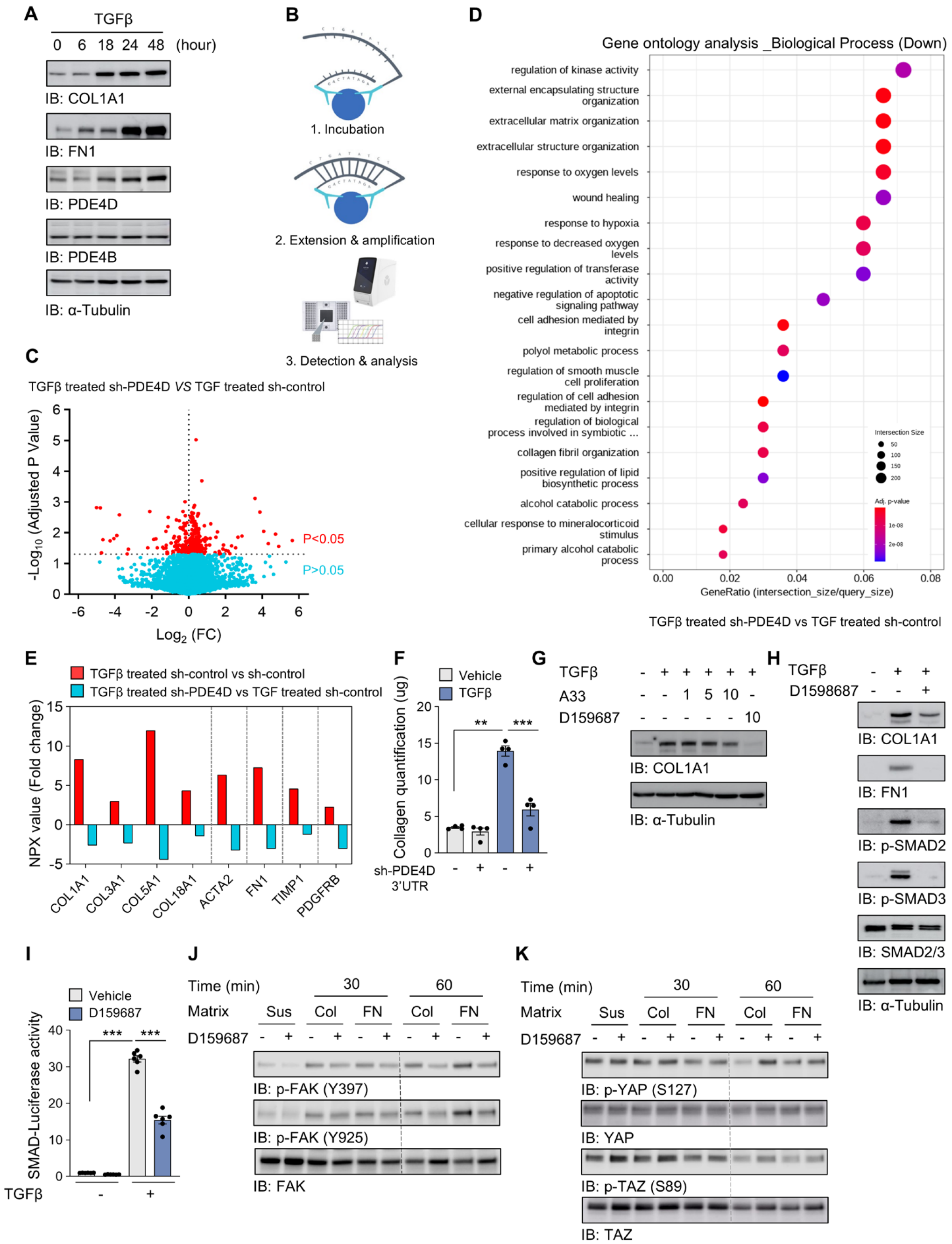

Supplemental Figure 4. Proteome-wide analysis between WT and PDE4D knockdown HSC. (A) PDE4D expression levels were measured after treatment with 2 ng/ml TGFβ for indicating times. **(B)** Schematic diagram of Proximity Extension Assay (PEA) in combination with Next-Generation Sequencing (NGS) readout for Olink proteomics. The PEA employs specifically paired

antibodies linked to distinct DNA oligonucleotides. Upon simultaneous attachment of these paired antibodies to a target protein, their associated DNA oligonucleotides undergo hybridization and establishing a distinctive barcode for NSG readout. The proteome-wide analysis was conducted on control and PDE4D knockdown cells following treatment with 2 ng/ml of TGFβ. **(C)** A volcano plot depicting the differentially expressed proteins in TGFβ-treated PDE4D knockdown HSC versus TGFβ-treated control HSCs. **(D)** Protein ontology analysis of biological process. **(E)** NPX value. Differential expressed protein in HSCs between sh-control and TGF-treated sh-control, or TGF-treated sh-control and TGF-treated sh-PDE4D. **(F)** Quantification of soluble collagen between WT and PDE4D knockdown HSC following treatment with 2 ng/ml of TGFβ. **(G)** LX-2 cells were treated with 2 ng/ml TGFβ for 24 hours in the presence of A33 and D159687. **(H)** LX-2 cells were incubated with TGFβ for 24 hours and subsequently treated with D159687 for 48 hours. **(I)** The transcriptional activity of SMAD was measured using reporter assay in smad-binding element (SBE)-stable reporter LX-2 cells. **(J, K)** LX-2 cells were pretreated with 10 μM D159687 for 24 and incubated on collagen or fibronectin-coated culture plates for the indicated duration. Phosphorylated FAK and YAP/TAZ levels were assessed by immunoblotting with antibodies specific for FAK Tyr397 or FAK Tyr 925 and YAP Ser127 or TAZ Ser89, respectively. All values are presented as the mean ± SEM of at least three independent experiments. One-way ANOVA with Tukey's post-hoc test for multiple comparisons was used for statistical analysis in F. $^{**}p < 0.01$; $^{***}p < 0.001$.

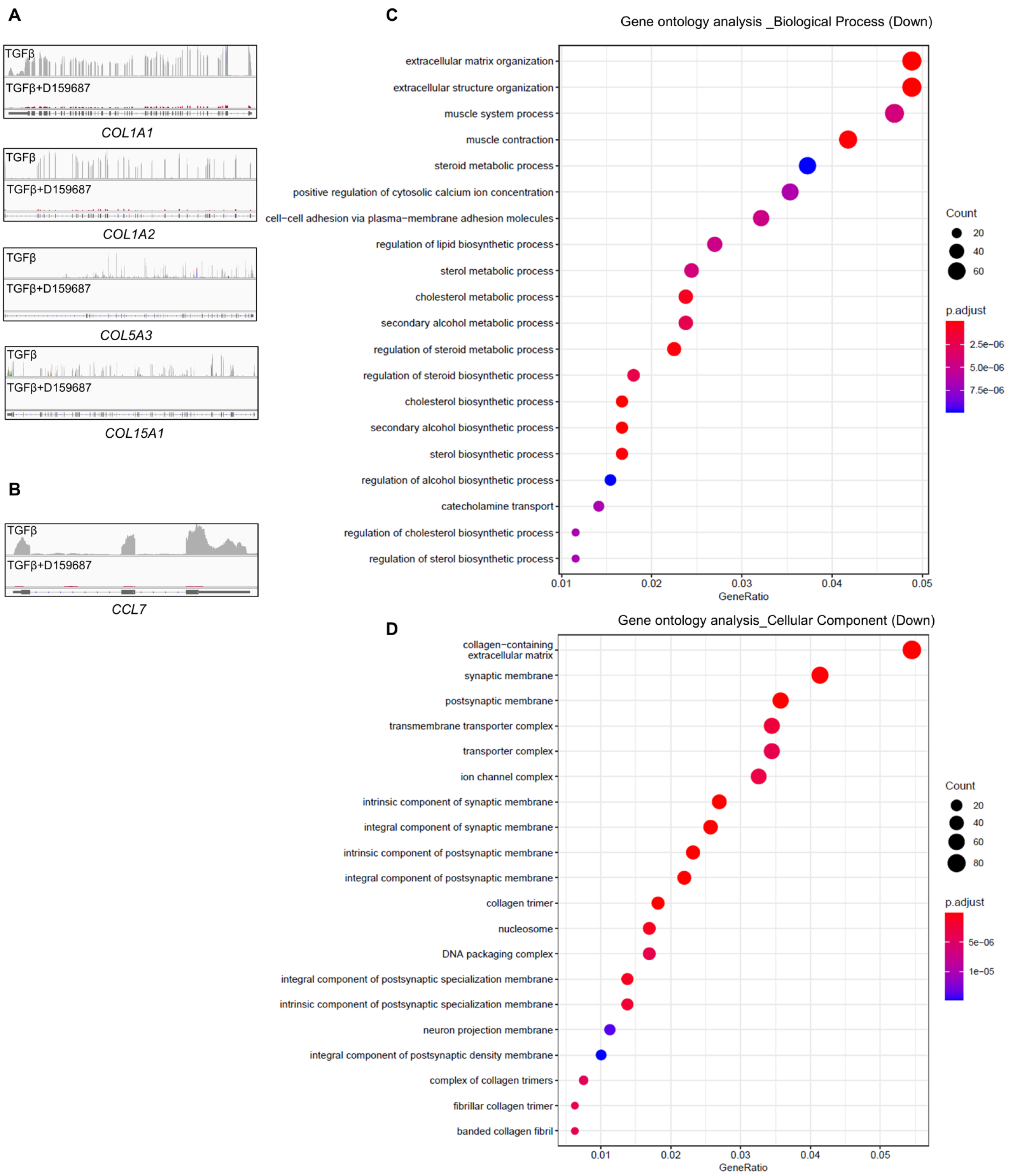

Supplemental Figure 5. Gene Ontology analysis of the RNA-seq data. (A, B) Coverage of RNA-seq of the collagen isoforms and *CCL7*. **(C, D)** Gene Ontology enrichment analysis of the down-regulated genes in LX-2 cells co-treated with TGFβ and D159687 compared with TGFβ-treated LX-2 cells, for biological processes and cellular components.

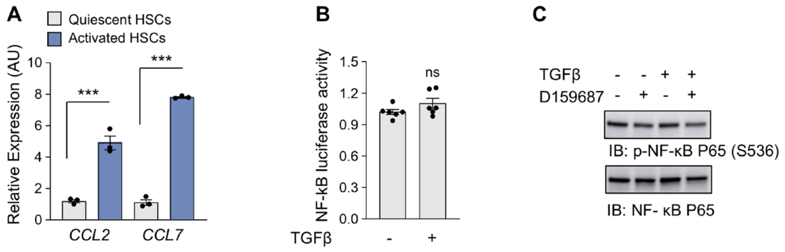

Supplemental Figure 6. Chemokines are upregulated in culture-activated HSCs. (A) Comparison of *CCL2* and *CCL7* mRNA levels between quiescent and culture-activated primary human HSCs were evaluated by RT-qPCR. **(B)** The transcriptional activity of NF-κB in stable reporter LX-2 cells with NF-κB response element was determined via a reporter assay. **(C)** Phosphorylated NF-κB P65 was visualized using an antibody specific for Ser536 and quantitatively compared with total NF-κB. All values are presented as the mean ± SEM of three independent experiments. A two-tailed unpaired Student's t-test was used to evaluate the statistical significance in A, B. ***$p < 0.001$; ns, not significant.

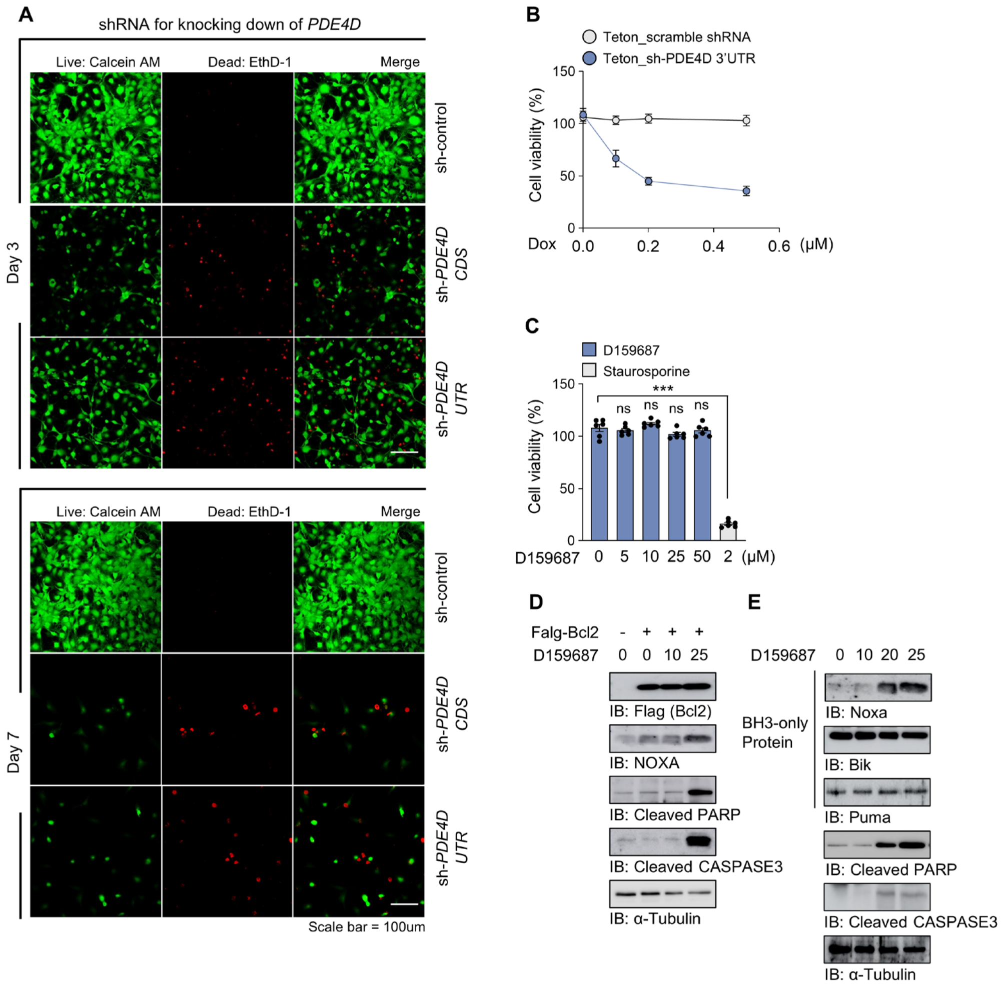

Supplemental Figure 7. Knocking down *PDE4D* causes growth retardation and apoptosis in LX-2 cells. (A) The viability of wild type and *PDE4D*-knockdown LX-2 cells were assessed via staining the cells with calcein-AM and ethidium homodimer-1 (EthD-1), which stain live and dead cells, respectively. **(B)** The cell viability was measured in HSCs that constitutively express Teton-scramble shRNA and teton-sh-PDE4D following treatment with doxycycline for 5 Days. **(C)** Viability of Kupffer cells treated with various concentrations of D159687. Staurosporine (2 μM) was used as a positive control of induction of apoptosis. **(D, E)** LX-2 cells overexpressing BCL-2 were treated with the indicated dose of D159687. One-way ANOVA with Tukey's post-hoc test for multiple comparisons was used for statistical analysis in B, C. ns, not significant.

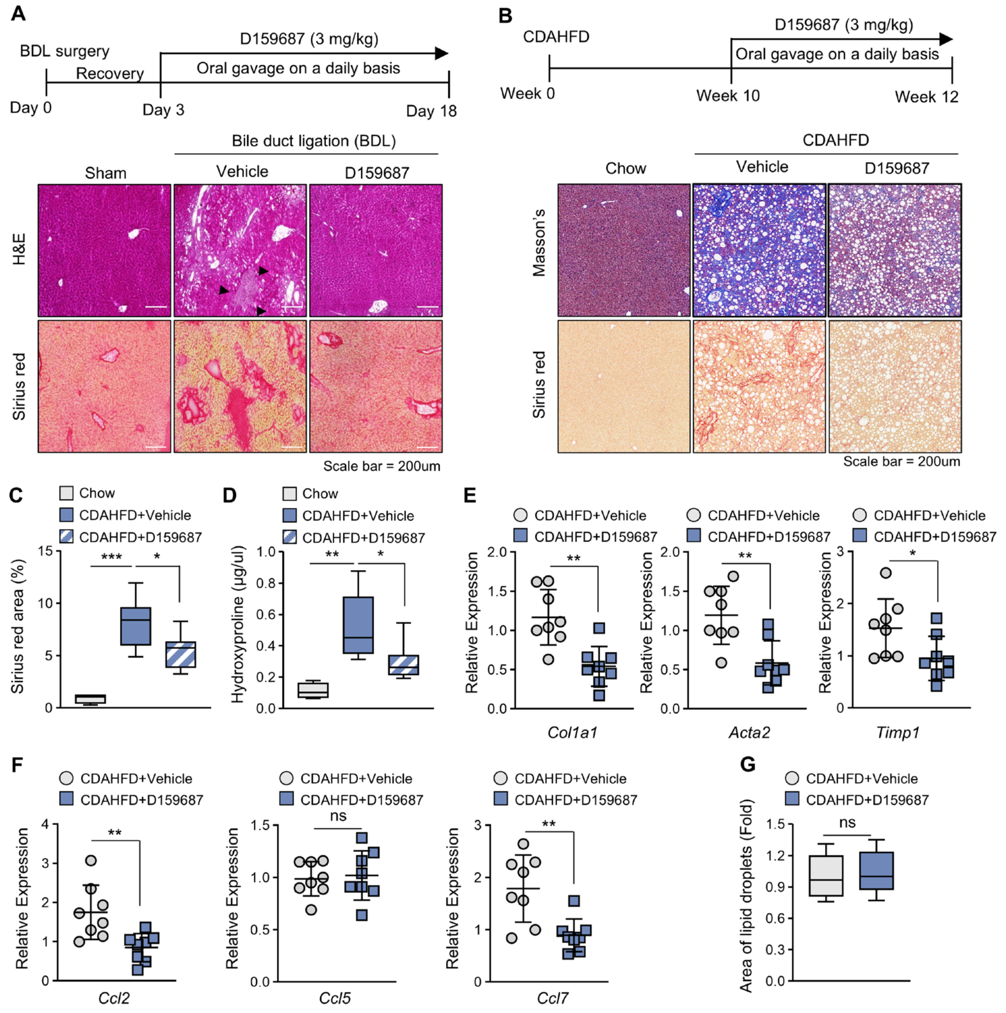

Supplemental Figure 8. The PDE4D allosteric inhibitor D159687 ameliorates the progression of liver fibrosis. (A) Representative images of mouse liver sections stained with Hematoxylin and eosin, or Sirius red (Sham-vehicle, n = 5; BDL-vehicle, n = 10; BDL-D159687, n = 10). BDL mice were daily treated with D159687 (3 mg/kg) or vehicle through oral gavage for 15 days after 3-day recovery from the surgery. **(B)** Representative images of mouse liver sections stained with masson's trichrome staining, or sirius red (chow diet-vehicle, n = 4; CDAHFD-vehicle, n = 8; CDAHFD-D159687, n = 8). Mice maintained on CDAHFD for 12 weeks were treated with D159687 (3 mg/kg) or vehicle by daily oral gavage from week 10 to week 12. (**C, D**) Quantification of the Sirius red-positive area and the level of hydroxyproline. (**E, F**) The mRNA

levels of the fibrotic markers Col1a1, Acta2, and Timp1; chemokines Ccl2, Ccl5, and Ccl7; in the livers of the mice were determined using RT-qPCR. (CDAHFD-vehicle, n = 8; CDAHFD-D159687, n = 8). (**G**) Analysis of the mean area of lipid droplets in liver tissue between CDAHFD-vehicle and CDAHFD-D159687 groups. All the values are presented as the mean ± SD. One-way ANOVA with Tukey's post-hoc test for multiple comparisons was used for statistical analysis in C and D; A two-tailed unpaired Student's t-test was used to evaluate the statistical significance in E to G. *$p < 0.05$; **$p < 0.01$; ns, not significant.

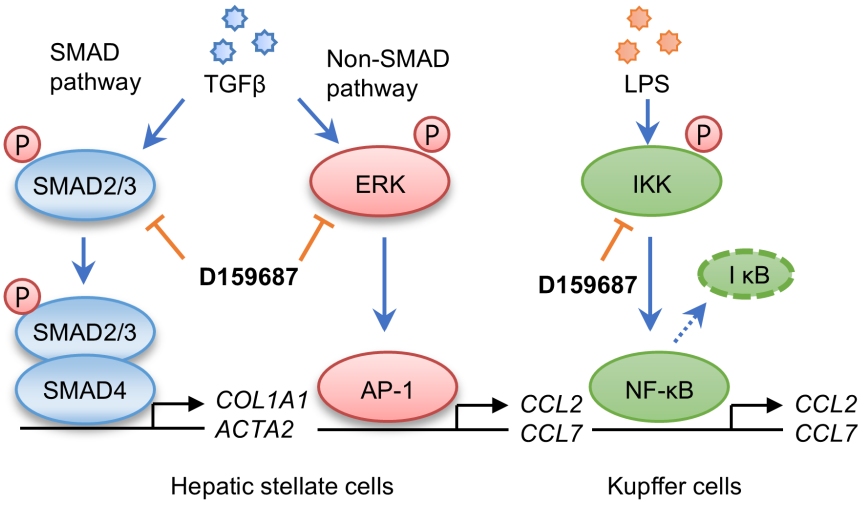

Supplemental Figure 9. Regulation of Fibrosis and Inflammatory Signaling by PDE4D. A schematic representation showing the signal-transduction pathways regulating profibrogenic and inflammatory signaling networks by PDE4D.

Supplemental Table 1. Sequences of short hairpin RNA.

Supplemental Table 1. Sequences of shRNAs

Gene Name	Targeted region	Target variants	Sequence
PDE4D	CDS	PDE4D1,PDE4D3,PDE4D5, PDE4D6, PDE4D7, PDE4D8, PDE4D9	CAGCTCTAGTCTGACTAATTC
	3'UTR	All	CCTAGTGTTTACCTGATTATA

Supplemental Table 2. Sequences of primers used for qPCR.

Supplemental Table 2. Sequence of primers for qRT-PCR

Gene Name	Forward Primer	Reverse Primer
mCol1a1	GGCAAACAAGGTCCTTCTGG	TCCCTCACGTCCAGATTCAC
hCOL1A1	GTACTGGATTGACCCCAACC	CGCCATACTCGAACTGGAAT
mActa2	CAATGTCCCCGCCATGTATG	CATCTCCAGAGTCCAGCACA
hACTA2	CCCCATCTATGAGGGCTATG	CAGTGGCCATCTCATTTTCA
mTimp1	TTCAAGGCTGTGGGAAATGC	CCACAGCCAGCACTATAGGT
hTIMP1	AGTGGCACTCATTGCTTGTG	GCAGGATTCAGGCTATCTGG
hSMAD7	TGTGCAAAGTGTTCAGGTGG	AGAGTCGGCTAAGGTGATGG
mCcl2	CCCAATGAGTAGGCTGGAGA	TCTGGACCCATTCCTTCTTG
hCCL2	TTGTGGCCAAGGAGATCTGT	TTTGGGTTTGCTTGTCCAGG
mCcl5	GTGCCCACGTCAAGGAGTAT	TCCTTCGAGTGACAAACACG
mCcl7	GATCTCTGCCACGCTTCTGT	ATAGCCTCCTCGACCCACTT
hCCL7	AGAGCTACAGAAGGACCACC	AGCACAGATCTCCTTGTCCA
mIl-β	CTGAACTCAACTGTGAAATGCCA	AAAGGTTTGGAAGCAGCCCT
mTNF-α	CCACCACGCTCTTCTGTCTA	CTGATGAGAGGGAGGCCATT
mNos2	CCAAGCCCTCACCTACTTCC	CTCTGAGGGCTGACACAAGG
mArg1	AAAGCTGGTCTGCTGGAAAA	ACAGACCGTGGGTTCTTCAC
mPde4d	ATCGTGAGTGGTACCAGAGC	CTCCACCTGACTTCCACTGT
hPDE4D	TGTGTGACAAGCACAATGCTTCC	CACGATTGTCCTCCAAAGTGTCCA
hPDE4D1	CCCCTTTGAACTCGCTAGC	TGCGAAGAGACAGGGAAAGG
hPDE4D5	AGACAAGCCCGGACACTTTA	GGGTTTGGACAATGCGGATT
mPde4b	GCTACAAGAGGAACACTGCG	CAGTTGCCAACACCATGTCA
hPDE4B	CTGGCCAAGGAGCTGGAA	AATATCCAGCCACATTAAAGATGTTAAG
mPde4c	CTCAGGAGCTTCTGGACACC	TCCAGGGTCAGCTCAAACTT
mGapdh	GACTTCAACAGCAACTCCCAC	TCCACCACCCTGTTGCTGTA
hGAPDH	GTCTCCTCTGACTTCAACAGCG	ACCACCCTGTTGCTGTAGCCAA
