# Supplementary material for: Selective inhibition of long isoforms of phosphodiesterase 4D mitigates liver fibrosis in mouse models
Source: J Clin Invest. 2025 Nov 6;136(1):e182571. doi: 10.1172/JCI182571 (PMC12721886; doi:10.1172/JCI182571)
Supplement: Unedited blot and gel images [file jci-136-182571-s214.pdf]

Full unedited gel for Figure 3D

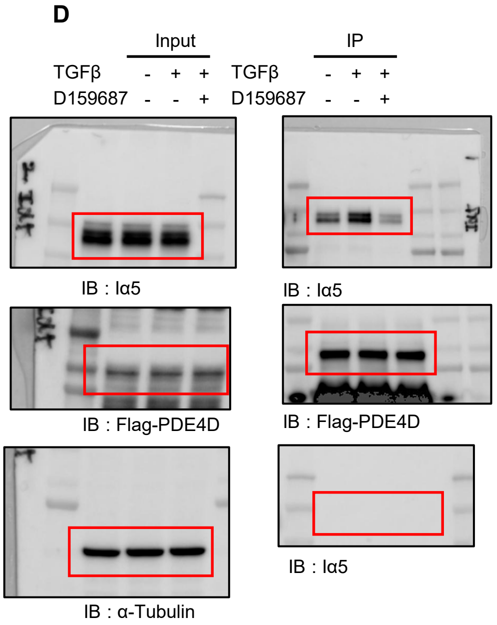

Full unedited gel for Figure 3E

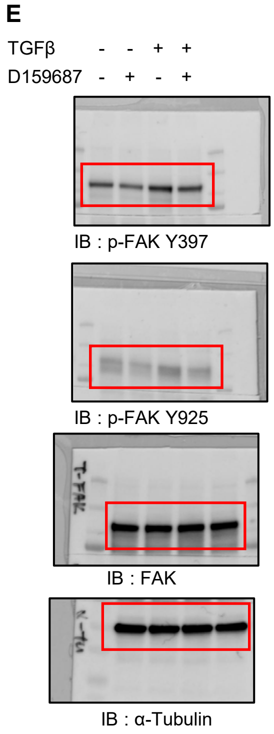

Full unedited gel for Figure 3F

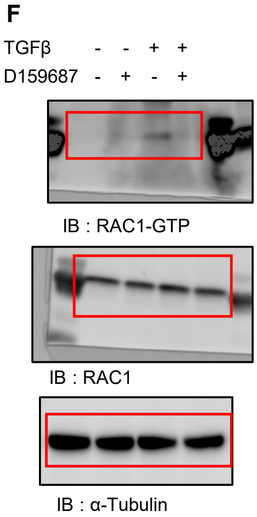

Full unedited gel for Figure 3G

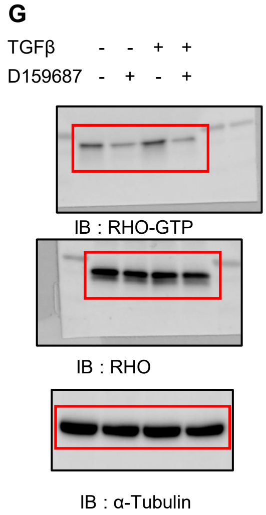

Full unedited gel for Figure 4B

B

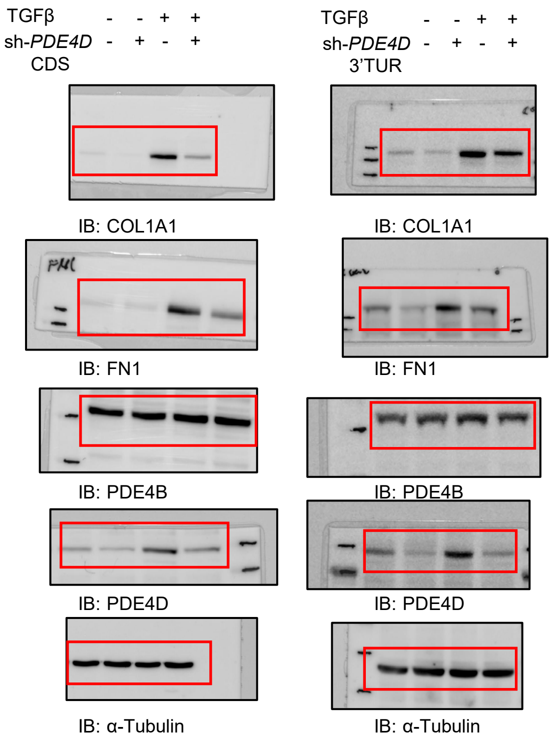

Full unedited gel for Figure 4E

E

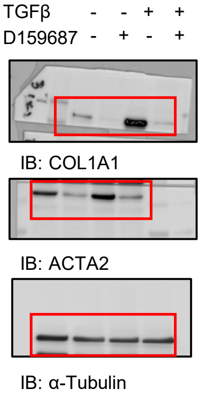

Full unedited gel for Figure 4F

F

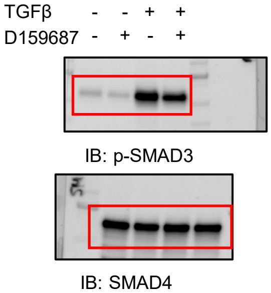

Full unedited gel for Figure 4H

H

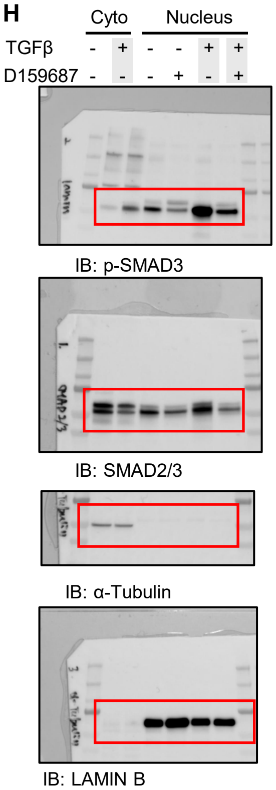

Full unedited gel for Figure 4I

I

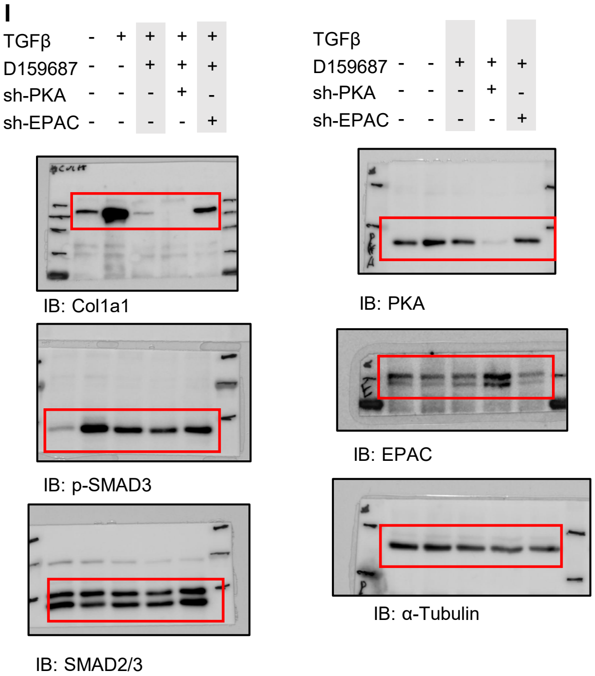

Full unedited gel for Figure 5F

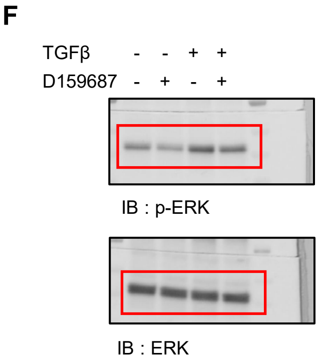

Full unedited gel for Figure 5J

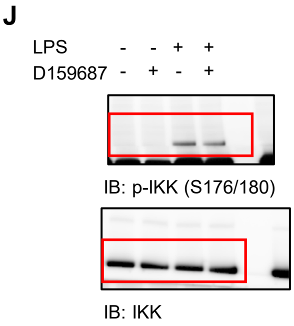

Full unedited gel for Figure 5K

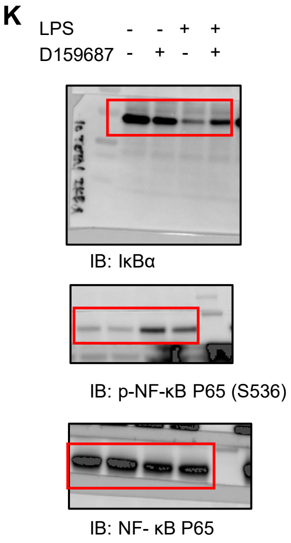

Full unedited gel for Figure 6B

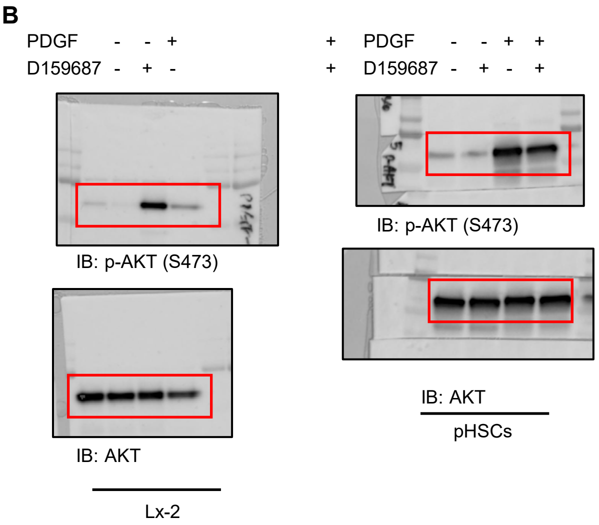

Full unedited gel for Figure 6C and D

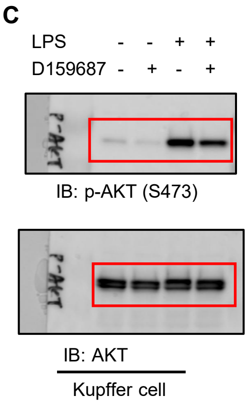

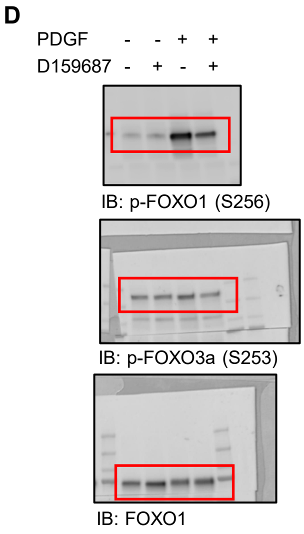

Full unedited gel for Figure 6F

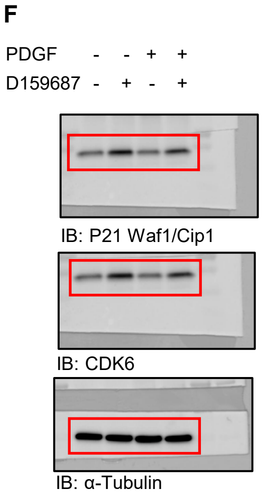

Full unedited gel for Figure 6I

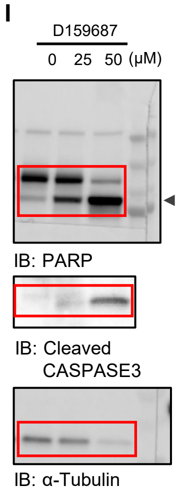

Full unedited gel for Figure S4A

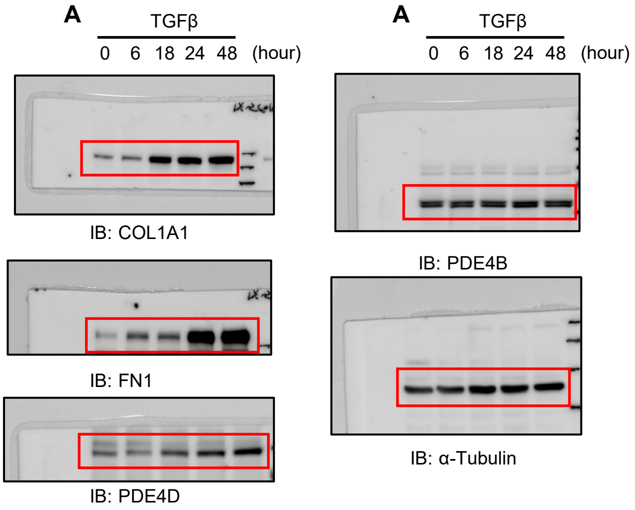

Full unedited gel for Figure S4G

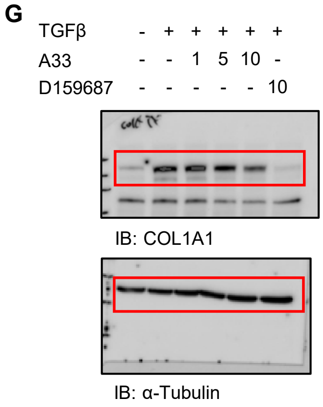

Full unedited gel for Figure S4H

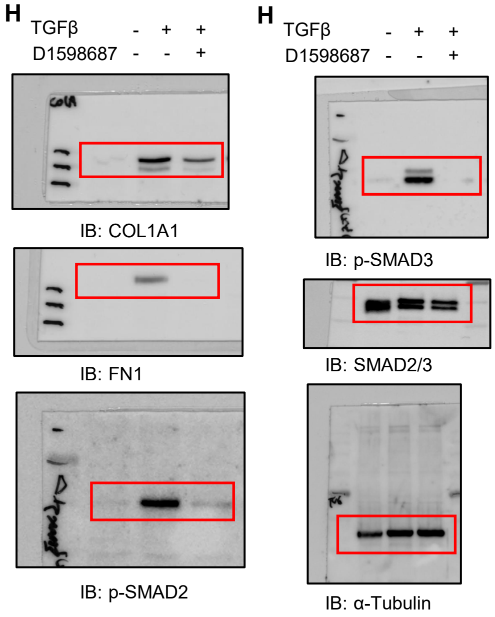

Full unedited gel for Figure S4J

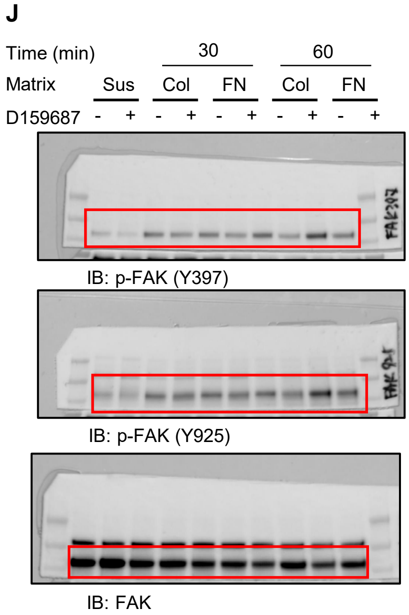

Full unedited gel for Figure S4K

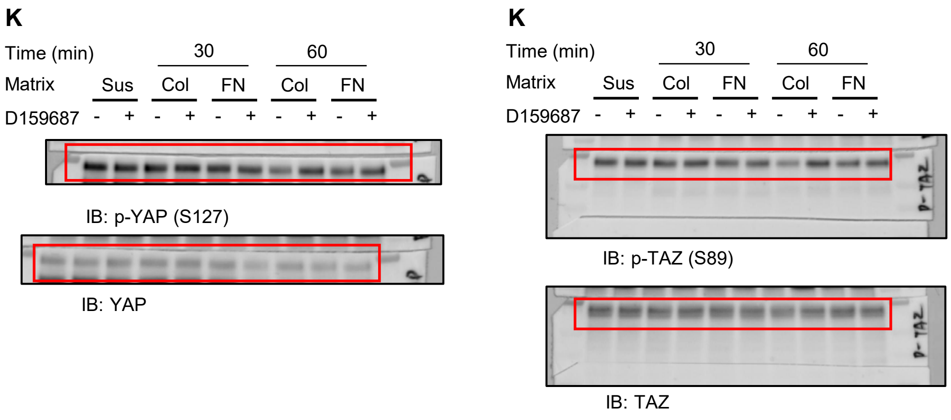

Full unedited gel for Figure S7D

D

Falg-Bcl2 - + + +
D159687 0 0 10 25

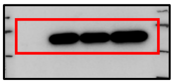

IB: Flag (Bcl2)

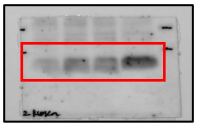

IB: NOXA

D

Falg-Bcl2 - + + +
D159687 0 0 10 25

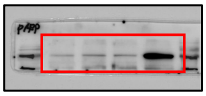

IB: Cleaved PARP

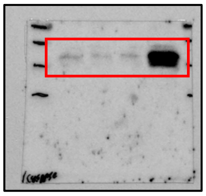

IB: Cleaved CASPASE3

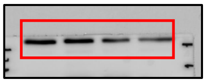

IB: α-Tubulin

Full unedited gel for Figure S7E

E

D159687 0 10 20 25

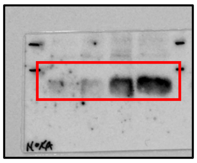

IB: Noxa

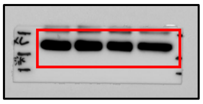

IB: Bik

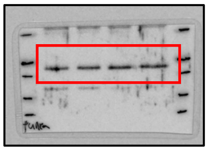

IB: Puma

E

D159687 0 10 20 25

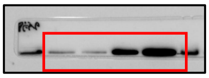

IB: Cleaved PARP

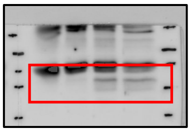

IB: Cleaved CASPASE3

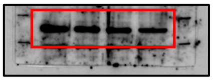

IB: α-Tubulin
